# Introducing neuromodulation in deep neural networks to learn adaptive behaviours

**DOI:** 10.1371/journal.pone.0227922

**Published:** 2020-01-27

**Authors:** Nicolas Vecoven, Damien Ernst, Antoine Wehenkel, Guillaume Drion

**Affiliations:** Department of Electrical Engineering and Computer Science Montefiore Institute, University of Liège, Liège, Belgium; SUNY Downstate MC, UNITED STATES

## Abstract

Animals excel at adapting their intentions, attention, and actions to the environment, making them remarkably efficient at interacting with a rich, unpredictable and ever-changing external world, a property that intelligent machines currently lack. Such an adaptation property relies heavily on *cellular neuromodulation*, the biological mechanism that dynamically controls intrinsic properties of neurons and their response to external stimuli in a context-dependent manner. In this paper, we take inspiration from cellular neuromodulation to construct a new deep neural network architecture that is specifically designed to learn adaptive behaviours. The network adaptation capabilities are tested on navigation benchmarks in a meta-reinforcement learning context and compared with state-of-the-art approaches. Results show that neuromodulation is capable of adapting an agent to different tasks and that neuromodulation-based approaches provide a promising way of improving adaptation of artificial systems.

## 1 Introduction

The field of machine learning has seen tremendous progress made during the past decade, predominantly owing to the improvement of deep neural network (DNN) algorithms. DNNs are networks of artificial neurons whose interconnections are tuned to reach a specific goal through the use of an optimization algorithm, mimicking the role of synaptic plasticity in biological learning. This approach has led to the emergence of highly efficient algorithms that are capable of learning and solving complex problems. Despite these tremendous successes, it remains difficult to learn models that generalise or adapt themselves efficiently to new, unforeseen problems based on past experiences. This calls for the development of novel architectures specifically designed to enhance adaptation capabilities of current DNNs.

In biological nervous systems, adaptation capabilities have long been linked to neuromodulation, a biological mechanism that acts in concert with synaptic plasticity to tune neural network functional properties. In particular, cellular neuromodulation provides the ability to continuously tune neuron input/output behaviour to shape their response to external stimuli in different contexts, generally in response to an external signal carried by biochemicals called neuromodulators [1, 2]. Neuromodulation regulates many critical nervous system properties that cannot be achieved solely through synaptic plasticity [3, 4]. It has been shown as being critical to the adaptive control of continuous behaviours, such as in motor control, among others [3, 4]. In this paper, we introduce a new neural architecture specifically designed for DNNs and inspired from cellular neuromodulation, which we call NMN, standing for “Neuro-Modulated Network”.

At its core, the NMN architecture comprises two neural networks: a main network and a neuromodulatory network. The main network is a feed-forward DNN composed of neurons equipped with a parametric activation function whose parameters are the targets of neuromodulation. It allows the main network to be adapted to new unforeseen problems. The neuromodulatory network, on the other hand, dynamically controls the neuronal properties of the main network via the parameters of its activation functions. Both networks have different inputs: the neuromodulatory network processes feedback and contextual data whereas the main network is in charge of processing other inputs.

Our proposed architecture can be related to previous works on different aspects. In [5], the authors take inspiration from Hebbian plasticity to build networks with plastic weights, allowing them to tune their weights dynamically. In [6] the same authors extend their work by learning a neuromodulatory signal that dictates which and when connections should be plastic. Our architecture is also related to hypernetworks [7], in which a network’s weights are computed through another network. Finally, other recent works focused on learning fixed activation functions [8, 9].

## 2 NMN architecture

The NMN architecture revolves around the neuromodulatory interaction between the neuromodulatory and main networks. We mimic biological cellular neuromodulation [10] in a DNN by assigning the neuromodulatory network the task to tune the slope and bias of the main network activation functions.

**Fig 1:**
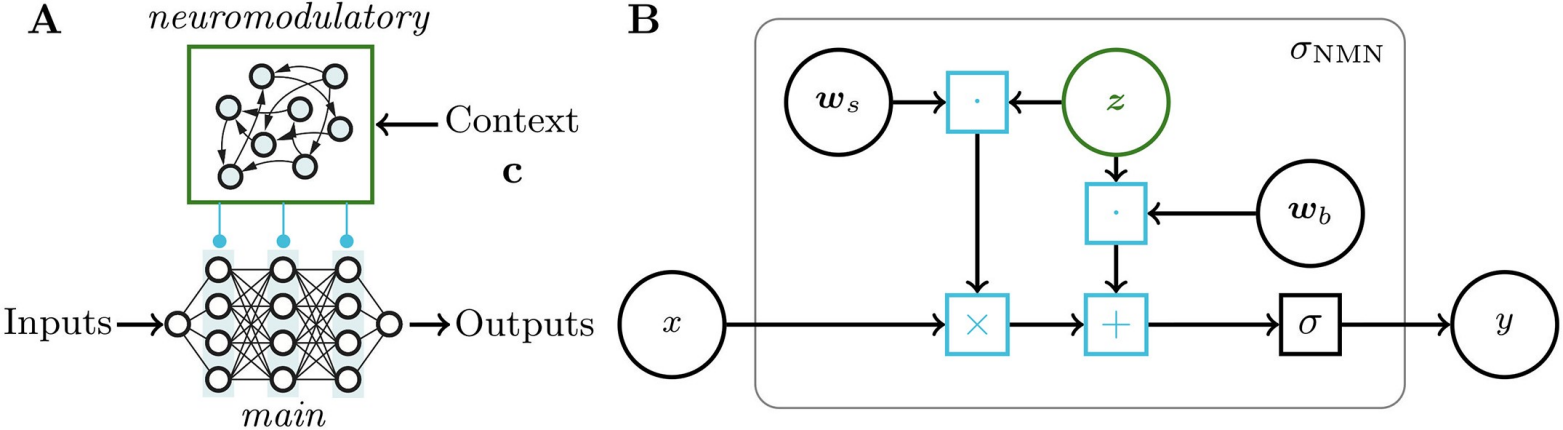
Sketch of the NMN architecture. **A**. The NMN is composed of the interaction of a *neuromodulatory* neural network that processes some context signal (top) and a *main* neural network that shapes some input-output function (bottom). **B**. Computation graph of the NMN activation functions *σ*_*NMN*_, where **w**_*s*_ and **w**_*b*_ are parameters controlling the scale factor and the offset of the activation function *σ*, respectively. **z** is a context-dependent variable computed by the neuromodulatory network.

Let σ(x):R→R denote any activation function and its neuromodulatory capable version *σ*_NMN_(*x*, **z**; **w**_*s*_, **w**_*b*_) = *σ*(**z**^*T*^(*x***w**_*s*_ + **w**_*b*_)) where $z\in R^{k}$ is a neuromodulatory signal and $w_{s},w_{b}\in R^{k}$ are two parameter vectors of the activation function, respectively governing a scale factor and an offset. In this work, we propose to replace all the main network neuron activation functions with their neuromodulatory capable counterparts. The neuromodulatory signal **z**, where size *k* is a free parameter, is shared for all these neurons and computed by the neuromodulatory network as **z** = *f*(**c**), where **c** is a vector representing contextual and feedback inputs. The function *f* can be any DNN taking as input such vector **c**. For instance, **c** may have a dynamic size (e.g. more information about the current task becomes available as time passes), in which case *f* could be parameterised as a recurrent neural network (RNN) or a conditional neural process [11], enabling refinement of the neuromodulatory signal as more data becomes available. The complete NMN architecture and the change made to the activation functions are depicted in Fig 1.

Notably, the number of newly introduced parameters scales linearly with the number of neurons in the main network whereas it would scale linearly with the number of connections between neurons if the neuromodulatory network was affecting connection weights, as seen for instance in the context of hypernetworks [7]. Therefore our approach can be extended more easily to very large networks.

## 3 Experiments

### 3.1 Setting

A good biologically motivated framework to which the NMN can be applied and evaluated is meta-reinforcement learning (meta-RL), as defined in [12]. In contrast with classical reinforcement learning (RL), which is formalised as the interaction between an agent and an environment defined as a Markov decision process (MDP), the meta-RL setting resides in the sub-division of an MDP as a distribution D over simpler MDPs. Let *t* denote the discrete time, **x**_*t*_ the state of the MDP at time *t*, **a**_*t*_ the action taken at time *t* and *r*_*t*_ the reward obtained at the subsequent time-step. At the beginning of a new episode *i*, a new element is drawn from D to define an MDP, referred to as M, with which the meta-RL agent interacts for T∈N time-steps afterwards. The only information that the agent collects on M is through observing the states crossed and the rewards obtained at each time-step. We denote by **h**_*t*_ = [**x**_0_, **a**_0_, *r*_0_, **x**_1_, …, **a**_*t*−1_, *r*_*t*−1_, **x**_*t*_] the history of the interaction with M up to time-step *t*. As in [12], the goal of the meta-learning agent is to maximise the expected value of the discounted sum of rewards it can obtain over all the time-steps and episodes.

### 3.2 Training

In [12], the authors tackle this meta-RL framework by using an advantage actor-critic (A2C) algorithm. This algorithm revolves around two distinct parametric functions: the actor and the critic. The actor represents the policy used to interact with the MDPs, while the critic is a function that rates the performance of the agent policy. All actor-critic algorithms follow an iterative procedure that consists of the three following steps.

1. Use the policy to interact with the environment and gather data.
2. Update the actor parameters using the critic ratings.
3. Update the critic parameters to better approximate a value function.

**Fig 2:**
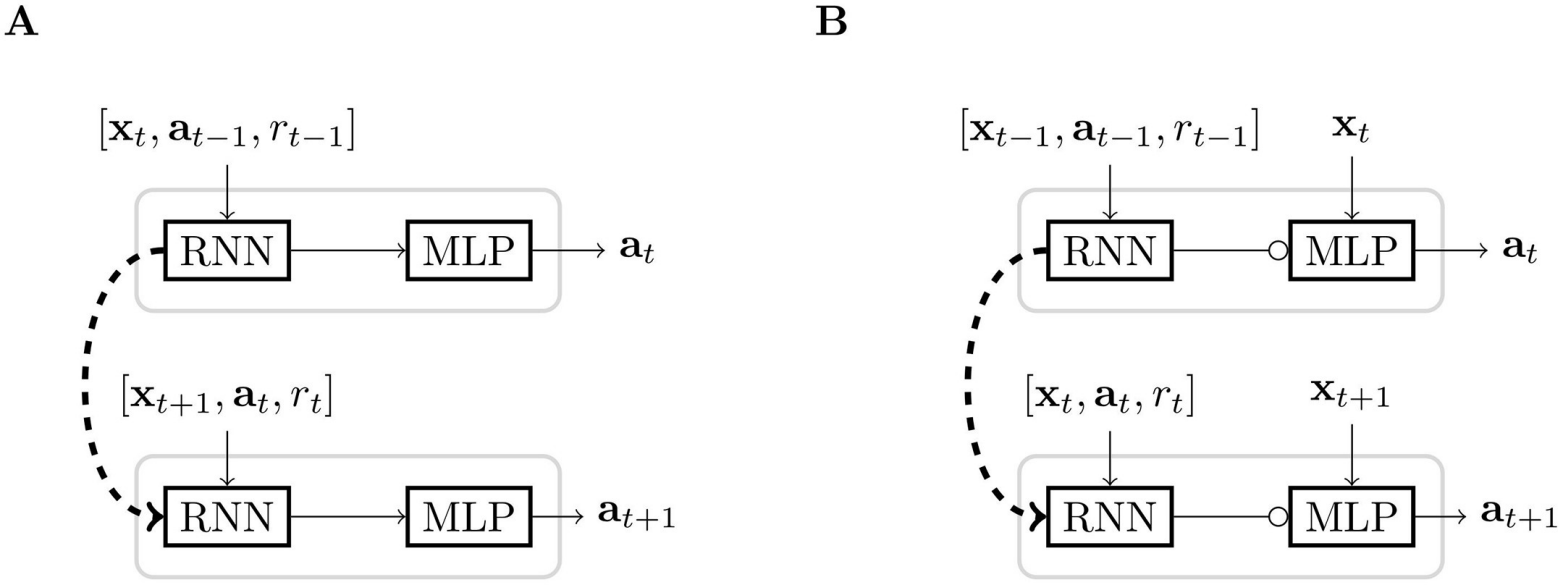
Sketch of a standard recurrent network (A) and of an NMN (B) in a meta-RL framework. → represent standard connections, ⊸ represent a neuromodulatory connection, ⇢ represent temporal connections and *MLP* stands for Multi-Layer Perceptron (standard feed-forward network).

In [12], the authors chose to model the actor and the critic with RNNs, taking **h**_*t*_ as the input. In this work, we propose comparing the NMN architecture to standard RNN by modelling both the actor and the critic with NMN. To this end, we define the feedback and contextual inputs **c** (i.e. the neuromodulatory network inputs) as **h**_*t*_\**x**_*t*_ while the main network input is defined as **x**_*t*_. Note that **h**_*t*_ grows as the agent interacts with M, motivating the usage of a RNN as neuromodulatory network. A graphical comparison between both architectures is shown on Fig 2.

To be as similar as possible to the neuronal model proposed by [10], the main network is a fully-connected neural network built using saturated rectified linear unit (sReLU) activation functions *σ*(*x*) = min(1, max(−1, *x*)), except for the final layer (also neuromodulated), for which *σ*(*x*) = *x*. In Section 4, we also report results obtained with sigmoidal activation functions which are often appreciably inferior to those obtained with sReLUs, further encouraging their use.

We built our models such that both standard RNN and NMN architectures have the same number of recurrent layers/units and a relative difference between the numbers of parameters that is lower than 2%. Both models are trained using an A2C algorithm with generalized advantage estimation [13] and proximal policy updates [14]. Finally, no parameter is shared between the actor and the critic. We motivate this choice by noting that the neuromodulatory signal might need to be different for the actor and the critic. For completeness and reproducibility, we provide a formal description of the algorithms used as supplementary material. This material aims mainly to describe and discuss standard RL algorithms in the context of meta-RL and, to a lesser extent, it aims to provide full implementation details. We also provide the exact neural architectures used for each benchmark as supplementary material.

### 3.3 Benchmarks description

**Fig 3:**
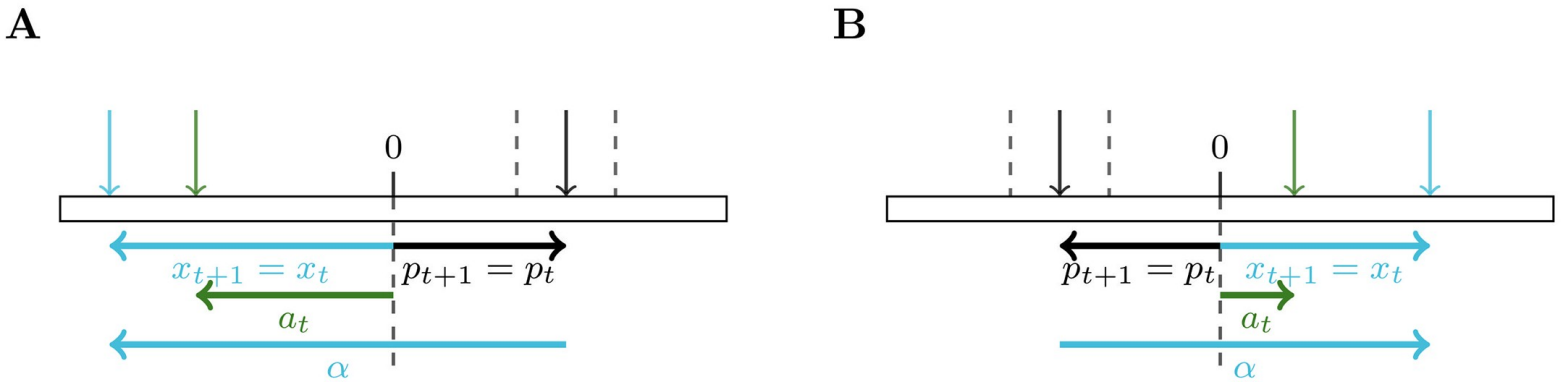
Sketch of a time-step interaction between an agent and two different tasks M (A and B) sampled in D for the first benchmark. Each task is defined by the bias *α* on the target’s position *p*_*t*_ observed by the agent. *x*_*t*_ is the observation made by the agent at time-step *t* and *a*_*t*_ its action. For these examples, *a*_*t*_ falls outside the target area (the zone delimited by the dashed lines), and thus the reward *r*_*t*_ received by the agent is equal to −|*a*_*t*_ − *p*_*t*_| and *p*_*t*+1_ = *p*_*t*_. If the agent had taken an action near the target, then it would have received a reward equal to 10 and the position of the target would have been re-sampled uniformly in [−5 − *α*, 5 − *α*].

**Fig 4:**
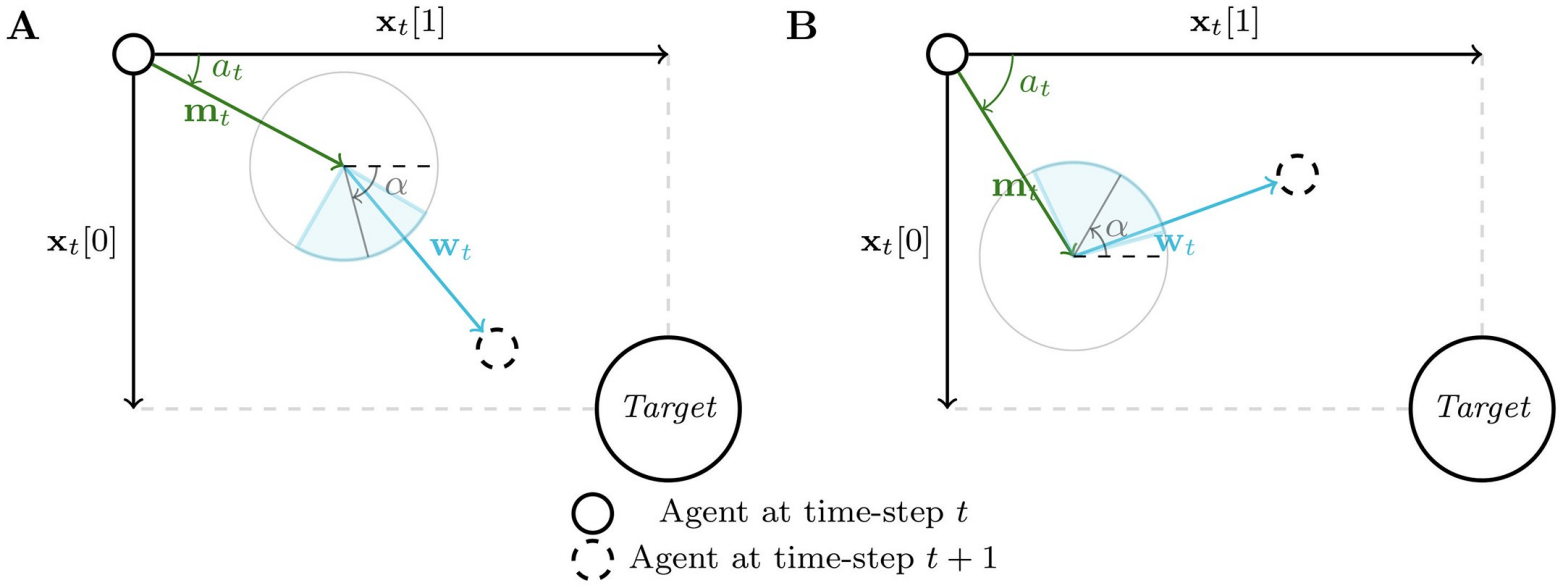
Sketch of a time-step interaction between an agent and two different tasks M (A and B) sampled in D for the second benchmark. Each task is defined by the main direction *α* of a wind cone from which a perturbation vector **w**_*t*_ is sampled at each time-step. This perturbation vector is then applied to the movement *m*_*t*_ of the agent, whose direction is given by the action *a*_*t*_. If the agent reaches the target, it receives a reward of 100, otherwise a reward of −2.

**Fig 5:**
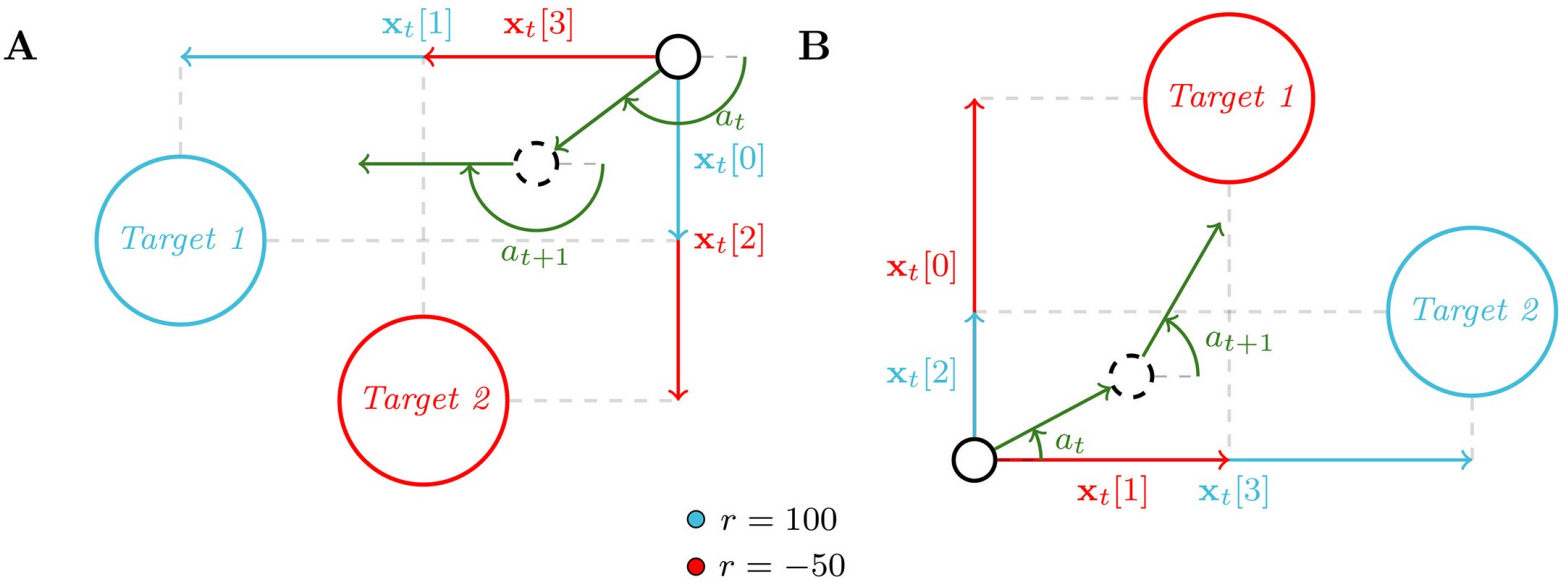
Sketch of a time-step interaction between an agent for the two different tasks M (A and B) sampled in D for the third benchmark. Each task is defined by the attribution of a positive reward to one of the two targets (in blue) and a negative reward to the other (in red). At each time-step the agent outputs an action *a*_*t*_ which drives the direction of its next move. If the agent reaches a target, it receives the corresponding reward.

We carried out our experiments on three custom benchmarks: a simple toy problem and two navigation problems with sparse rewards. These benchmarks were built to evaluate our architecture in environments with continuous action spaces. For conciseness, we only provide a mathematical definition of the first benchmark. The two other benchmarks are briefly textually depicted and further details are available as supplementary material. Figs 3, 4 and 5 are a graphical representation of each of the benchmarks.

#### Benchmark 1

We define the first benchmark (made of a 1-D state space and action space) through a random variable *α*, informative enough to distinguish all different MDPs in D. With this definition, *α* represents the current task and drawing *α* at the beginning of each episode amounts to sampling a new task in D. At each time-step, the agent observes a biased version *x*_*t*_ = *p*_*t*_ + *α* of the exact position of a target *p*_*t*_ belonging to the interval [−5 − *α*, 5 − *α*], with α∼U[-10,10]. The agent outputs an action *a*_*t*_ ∈ [−20, 20] and receives a reward *r*_*t*_ which is equal to 10 if |*a*_*t*_ − *p*_*t*_| < 1 and −|*a*_*t*_ − *p*_*t*_| otherwise. In case of positive reward, *p*_*t*+1_ is re-sampled uniformly in its domain, else *p*_*t*+1_ = *p*_*t*_. This benchmark is represented on Fig 3.

#### Benchmark 2

The second benchmark consists of navigating towards a target in a 2-D space with noisy movements. Similarly to the first benchmark, we can distinguish all different MDPs in D through a three-dimensional random vector of variables ***α***. The target is placed at (***α***[1], ***α***[2]) in the 2-D space. At each time-step, the agent observes its relative position to the target and outputs the direction of a move vector **m**_*t*_. A perturbation vector **w**_*t*_ is then sampled uniformly in a cone, whose main direction α[3]∼U[-π,π[, together with the target’s position, define the current task in D. Finally the agent is moved following **m**_*t*_ + **w**_*t*_ and receives a reward (*r*_*t*_ = −0.2). If the agent reaches the target, it instead receives a high reward (*r*_*t*_ = 100) and is moved to a position sampled uniformly in the 2-D space. This benchmark is represented on Fig 4.

#### Benchmark 3

The third benchmark also involves navigating in a 2-D space, but which contains two targets. As for the two previous benchmarks, we distinguish all different MDPs in D through a five-dimensional random vector of variables ***α***. The targets are placed at positions (***α***[1], ***α***[2]) and (***α***[3], ***α***[4]). At each time-step, the agent observes its relative position to the two targets and is moved along a direction given by its action. One target, defined by the task in D through ***α***[5], is attributed a positive reward (100) and the other a negative reward (−50). In other words, ***α***[5] is a Bernoulli variable that determines which target is attributed the positive reward and which is attributed the negative one. As for benchmark 2, once the agent reaches a target, it receives the corresponding reward and is moved to a position sampled uniformly in the 2-D space. This benchmark is represented on Fig 5.

## 4 Results

### Learning

**Fig 6:**
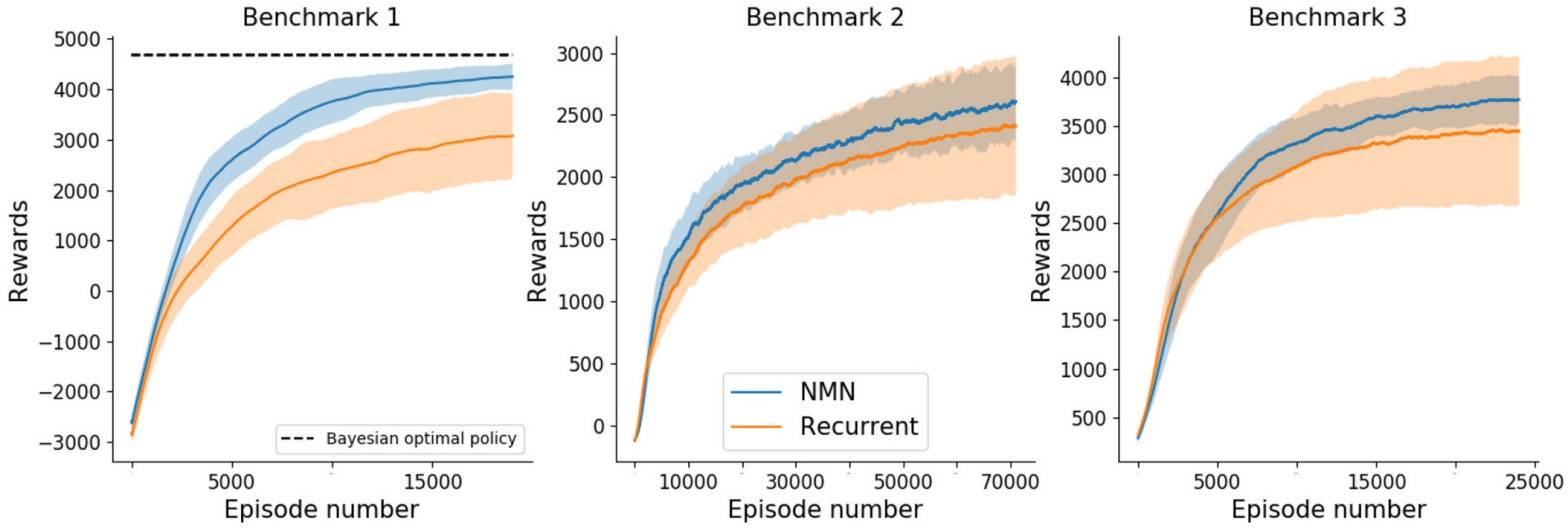
Mean (± std in shaded) sum of rewards obtained over 15 training runs with different random seeds with respect to the episode number. Results of benchmark 1,2 and 3 are displayed from left to right. The plots are smoothed thanks to a running mean over 1000 episodes.

From a learning perspective, a comparison of the sum of rewards obtained per episode by NMNs and RNNs on the three benchmarks is shown in Fig 6. Results show that, on average, NMNs learn faster (with respect to the number of episodes) and converge towards better policies than RNNs (i.e., higher rewards for the last episodes). It is worth mentioning that, NMNs show very stable results, with small variances over different random seeds, as opposed to RNNs. To put the performance of the NMN in perspective, we note that an optimal Bayesian policy would achieve an expected sum of rewards of 4679 on benchmark 1 (see supplementary material for proof) whereas NMNs reach, after 20000 episodes, an expected sum of rewards of 4534. For this simple benchmark, NMNs manage to learn near-optimal Bayesian policies.

### Adaptation

**Fig 7:**
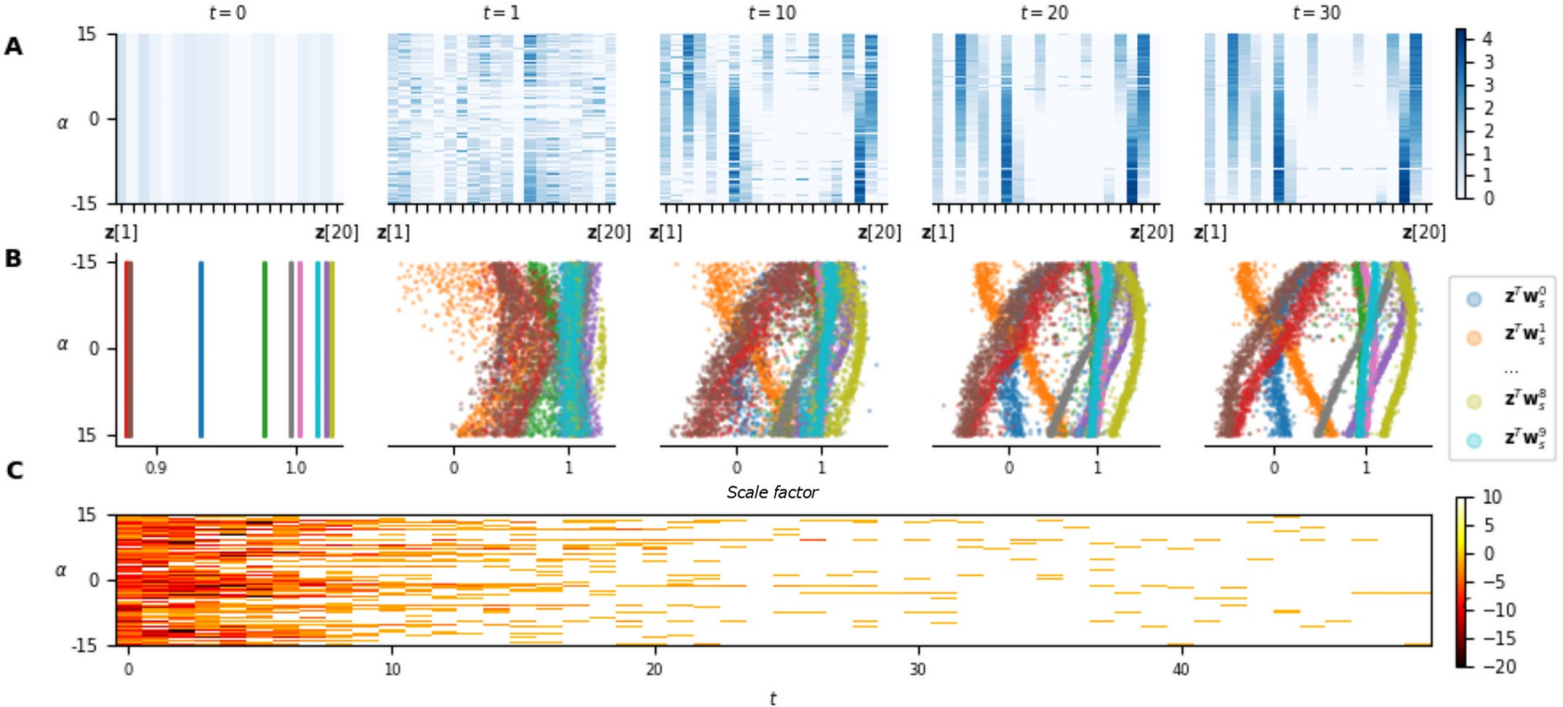
Adaptation capabilities of the NMN architecture on benchmark 1. **A**. Temporal evolution of the neuromodulatory signal **z** with respect to *α*, gathered on 1000 different episodes. Note that the neuromodulatory signals go from uniform distributions over all possible *α* values (i.e., the different contexts) to non-uniform and adapted (w.r.t. *α*) distributions along with an increase in the rewards. **B**. The value of the scale factors with respect to *α* for each neuron of a hidden layer in the main network. **C**. Rewards obtained at each time-step by the agent during those episodes. Note that light colours represent high rewards and correspond to adapated neuromodulatory signals.

From an adaptation perspective, Fig 7 shows the temporal evolution of the neuromodulatory signal **z** (part **A**), of the scale factor (for each neuron of a hidden layer, part **B**) and of the rewards (part **C**) obtained with respect to *α* for 1000 episodes played on benchmark 1. For small values of *t*, the agent has little information on the current task, leading to a non-optimal behaviour (as it can be seen from the low rewards). Of greatest interest, the signal **z** for the first time-steps exhibits little dependence on *α*, highlighting the agent uncertainty on the current task and translating to noisy scale factors. Said otherwise, for small *t*, the agent learned to play a (nearly) task-independent strategy. As time passes, the agent gathers further information about the current task and approaches a near-optimal policy. This is reflected in the convergence of **z** (and thus scale factors) with a clear dependency on *α* and also in wider-spread values of **z**. For a large value of *t*, **z** holding constant between time-steps shows that the neuromodulatory signal is almost state-independent and serves only for adaptation. We note that the value of **z** in each of its dimensions varies continuously with *α*, meaning that for two similar tasks, the signal will converge towards similar values. Finally, it is interesting to look at the neurons scale factor variation with respect to *α* (**B**). Indeed, for some neurons, one can see that the scale factors vary between negative and positive values, effectively inverting the slope of the activation function. Furthermore, it is interesting to see that some neurons are inactive (scale factor almost equal to 0, leading to a constant activation function) for some values of *α*.

**Fig 8:**
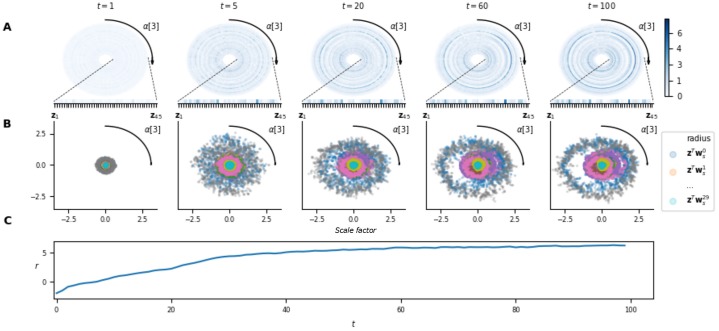
Adaptation capabilities of the NMN architecture on benchmark 2. **A**. Temporal evolution of the neuromodulatory signal **z** with respect to ***α***[3], gathered on 1000 different episodes. As ***α***[3] is an angle, the plot is projected in polar coordinates for a better interpretability of the results. Each dimension of **z** is corresponds to a different radius. **B**. The value of the scale factors with respect to ***α***[3] for each neuron of a hidden layer in the main network. Again, the plot is projected in polar coordinates. For a given ***α***[3], the values of the neurons’ scale factor are given thanks to the radius. **c**. Average reward obtained at each time-step by the agent during those episodes. Note that after an average of 40 time-steps, the agent is already achieving decent performances even though **z** has not yet converged.

For benchmark 2, let us first note that **z** seems to code exclusively for ***α***[3]. Indeed, **z** converges slowly with time with respect to ***α***[3], whatever the value of ***α***[1] and ***α***[2] (Fig 8). This, could potentially be explained by the fact that one does not need the values of ***α***[1] and ***α***[2] to compute an optimal move. The graphs on Fig 8 are projected on the dimension ***α***[3], allowing the same analysis as for benchmark 1.

The results obtained for benchmark 2 (Fig 8) show similar characteristics. Indeed, despite the agent receiving only noisy information on ***α***[3] at each time-step (as perturbation vectors are sampled uniformly in a cone centered on ***α***[3]), **z** quasi-converges slowly with time (part **A**). The value of **z** in each of its dimensions also varies continuously with ***α***[3] (as for the first benchmark) resulting also in continuous scale factors variations. This is clearly highlighted at time-step 100 on Fig 8 where the scale factors of some neurons appear highly asymmetric, but with smooth variations with respect to ***α***[3]. Finally, let us highlight that for this benchmark, the agent continues to adapt even when it is already performing well. Indeed, one can see that after 40 time-steps the agent is already achieving good results (part **C**), even though **z** has not yet converged (part **A**), which is due to the stochasticity of the environment. Indeed, the agent only receives noised information on *α* and thus after 40 time-steps it has gathered sufficient information to act well on the environment, but insufficient information to deduce a near-exact value of ***α***[3]. This shows that the agent can perform well, even while it is still gathering relevant information on the current task.

**Fig 9:**
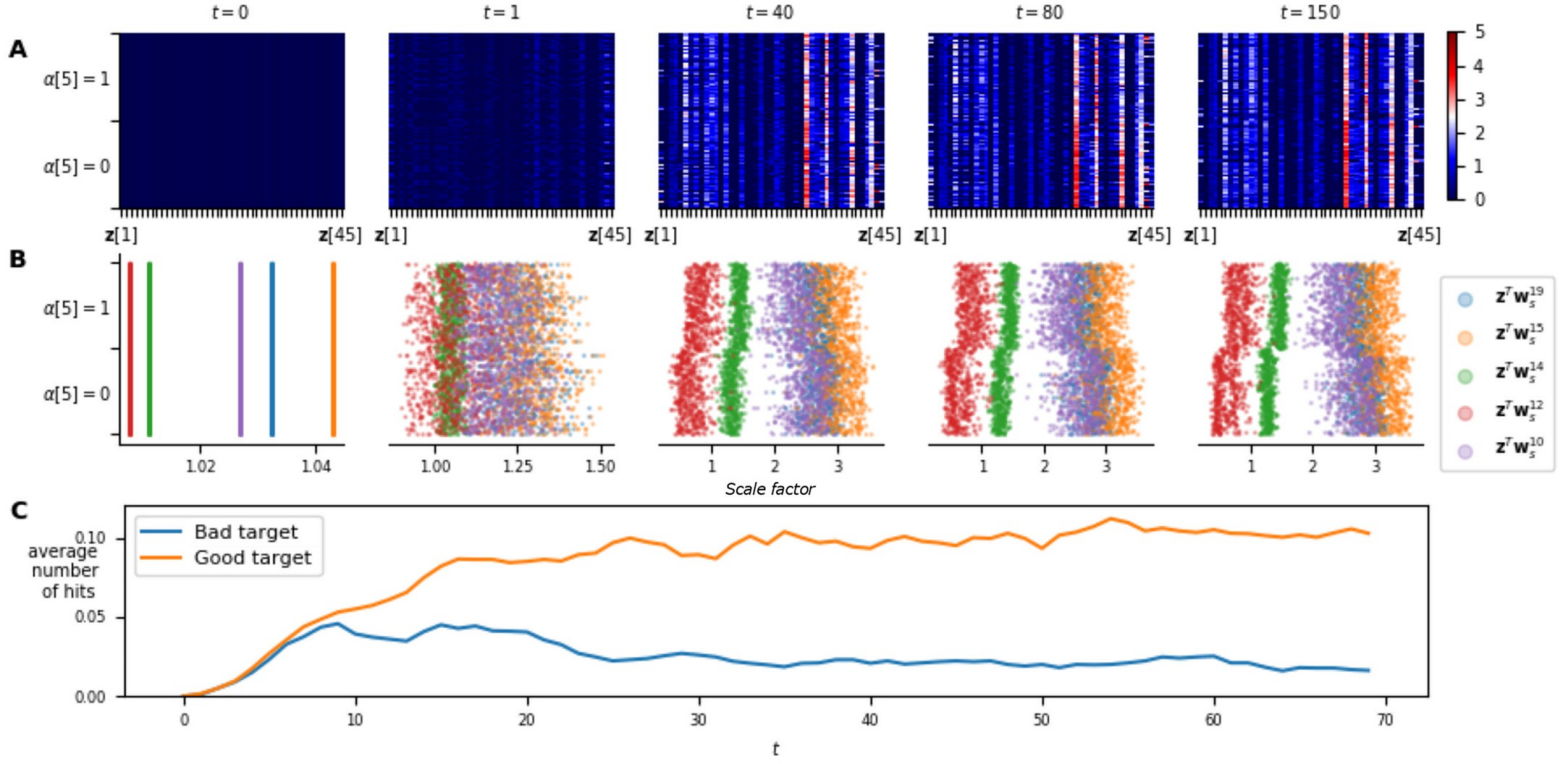
Adaptation capabilities of the NMN architecture on benchmark 3. **A**. Temporal evolution of the neuromodulatory signal **z** with respect to ***α***[5], gathered on 1000 different episodes. Note that the neuromodulatory signals go from uniform distributions over all possible alpha values (i.e., the different contexts) to non-uniform and adapted (w.r.t. alpha) distributions along with an increase of the rewards. **B**. The value of the scale factors with respect to ***α***[5] for the 5 neurons of a hidden layer in the main network, for which the scale factor is the most correlated to ***α***[5]. **C**. Average number of good and bad target hits at each time-step during those episodes. On average, after 15 time-steps, the agent starts navigating towards the correct target while avoiding the wrong one.

It is harder to interpret the neuromodulatory signal for benchmark 3. In fact, for that benchmark, we show that the signal seems to code not only for the task in D but also for the state of the agent in some sense. As ***α*** is five-dimensional, it would be very difficult to look at its impact on **z** as a whole. Rather, we fix the position of the two references in the 2-D space and look at the behaviour of **z** with respect to ***α***[5]. In Fig 9 adaptation is clearly visible in the rewards obtained by the agent (part **C**) with very few negative rewards after 30 time-steps. We note that for later time-steps, **z** tends to partially converge (**A**) and:

- some dimensions of **z** are constant with respect to ***α***[5], indicating that they might be coding for features related to ***α***[1, 2, 3, 4].
- Some other dimensions are well correlated to ***α***[5], for which similar observations than for the two other benchmarks can be made. For example, one can see that some neurons have a very different scale factors for the two possible different values of ***α***[5] (**B**).
- The remaining dimensions do not converge at all, implying that these are not related to ***α***, but rather to the state of the agent.

**Fig 10:**
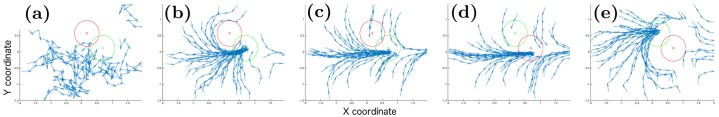
Analysis of the agent’s behaviour when freezing and unfreezing the neuromodulation signal and when changing task within an episode. The green reference is attributed a reward of 100 while the red one is attributed a reward of −50. Each blue arrow represents the movement of the agent for a given time-step. **(a)** Shows the behaviour with **z** fixed at its initial value. In **(b)** we unlock **z**. Then, in **(c)** we lock **z** with its current value. Finally in **(d)** we switch the references before unlocking **z** once again in **(e)**.

These results suggest that in this case, the neuromodulation network is used to code more complex information than simply that required to differentiate tasks, making **z** harder to interpret. Despite **z** not converging on some of its dimensions, we stress that freezing **z** after adaptation will not strongly degrade the agent’s performance. That is, the features coded in **z** that do not depend on ***α*** are not critical to the performance of the agent. To illustrate this, we will analyse the behaviour of the agent within an episode when freezing and unfreezing the neuromodulation signal and when changing task. This behaviour is shown on Fig 10, for which:

(a) Shows the behaviour of the agent when **z** is locked to its initial value. This plot thus shows the initial “exploration” strategy used by the agent; that is, the strategy played by the agent when it has not gathered any information on the current task.
(b) Shows the behaviour of the agent after unlocking **z**, that is when the agent is able to adapt freely to the current task by updating **z** at each time-step.
(c) Shows the behaviour of the agent when locking **z** at a random time-step after adaptation. **z** is thus fixed at a value which fits well the current task. As one can see, the agent continues to navigate towards the correct target. The performance is however a slightly degraded as the agent seems to lose some capacity to avoid the wrong target. This further suggests that, in this benchmark (as opposed to the two others), the neuromodulation signal does not only code for the current task but also for the current state, in some sense, that is hard to interpret.
(d) Shows the same behaviour as in **(c)** as **z** is still locked to the same value, but the references are now switched. As there is no adaptation without updating **z**; the agent is now always moving towards to wrong target.
(e) Shows the behaviour of the agent when unlocking **z** once again. As one can see, the agent is now able to adapt correctly by updating **z** at each time-step, and thus it navigates towards the correct target once again.

### Robustness study

Even though results are quite promising for the NMN, it is interesting to see how it holds up with another type of activation function as well as analysing its robustness to different main networks’ architectures.

### Sigmoid activation functions

**Fig 11:**
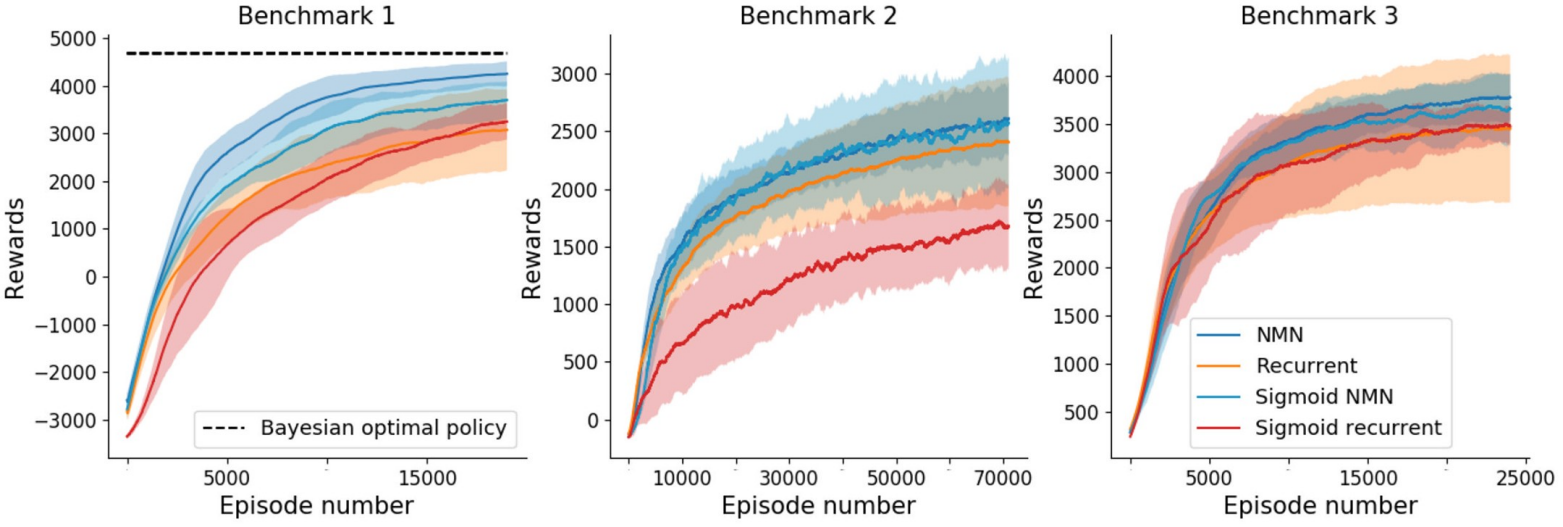
Mean (± std in shaded) sum of rewards obtained over 15 training runs with different random seeds with respect to the episode number. Results of benchmark 1, 2 and 3 are displayed from left to right. The plots are smoothed thanks to a running mean over 1000 episodes.

Fig 11 shows the comparison between having sigmoids as the main network’s activation function instead of sReLUs. As one can see, sigmoid activation functions lead to worse or equivalent results to sReLUs, be it for RNNs or NMNs. In particular, the NMN architecture seems more robust to the change of activation function as opposed to RNNs, as the difference between sReLUS and sigmoids is often far inferior for NMNs than RNNs (especially for benchmark 2).

### Architecture impact

**Fig 12:**
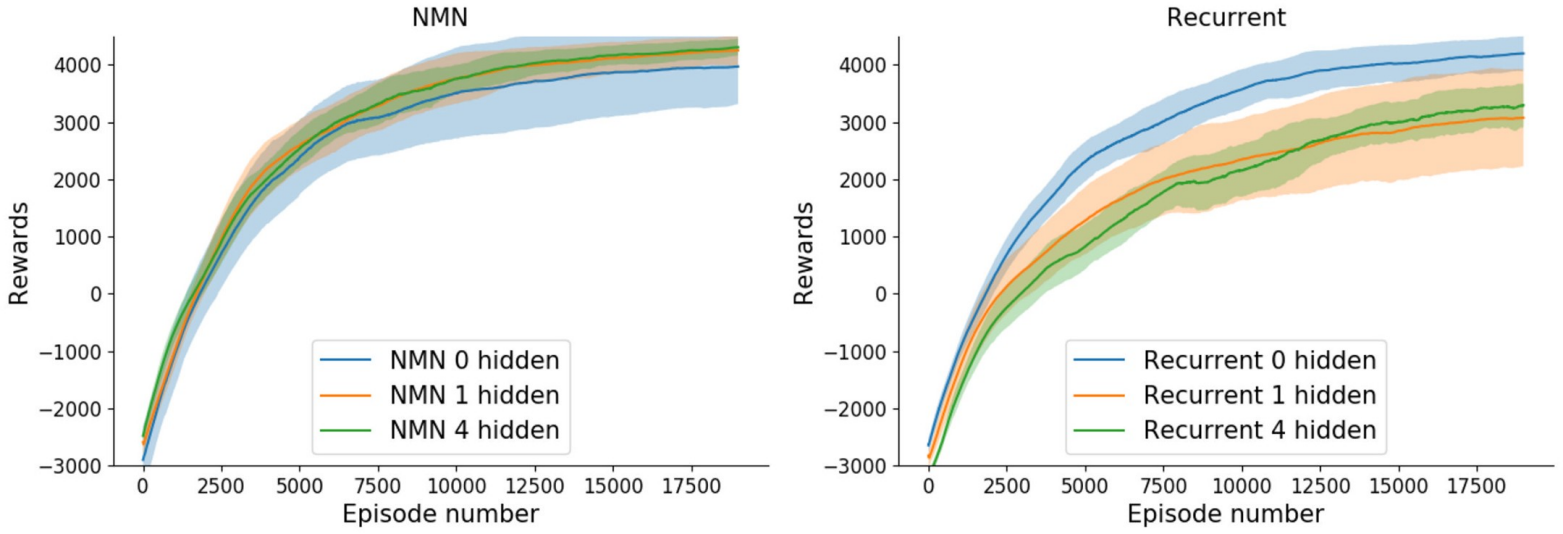
Mean (± std in shaded) sum of rewards obtained on benchmark 1 over 15 training runs with different random seeds with respect to the episode number. The plots are smoothed thanks to a running mean over 1000 episodes.

Fig 12 shows the learning curve, on benchmark 1, for different main network architectures (0, 1 and 4 hidden layers in the main network respectively). As one can see, RNNs can, in fact, reach NMNs’ performances for a given architecture (no hidden layer in this case), but seem relatively dependant on the architecture. On the contrary, NMNs seem surprisingly consistent with respect to the number of hidden layers composing the main network.

## 5 Conclusions

In this work, we adopt a high-level view of a nervous system mechanism called cellular neuromodulation in order to improve the adaptive capabilities of artificial neural networks. The results obtained for three meta-RL benchmark problems showed that this new architecture was able to perform better than classical RNN. The work reported in this paper could be extended along several lines.

First, it would make sense to explore other types of machine-learning problems where adaptation is required. Supervised meta-learning would be an interesting track to follow as, for example, it is easy to see how our architecture could be applied to few-shot learning. In such a framework, the context fed to the neuromodulatory network would be a set composed of a few samples and their associated ground-truth. It would be of great interest to compare the performance of our architecture to that of conditional neural processes [11] (CNP). Indeed, the NMN used in this few-shot setting can, in fact, be seen as a CNP with a specifically designed neuromodulatory connection for conditioning the main network.

Second, research work could also be carried out to further improve the NMN introduced here. For instance, one could introduce new types of parametric activation functions which are not linear, or even spiking neurons. This would amount to designing a brand-new parametric activation functions, the parameters of which could thus be more powerful than simple slope and bias. It would also be of interest to look at sharing activation function parameters per layer, especially in convolution layers, as this would essentially result in scaling the filters. One could also build a neuromodulatory signal per-layer rather than for the whole network, allowing for more complex forms of modulation. Furthermore, it would be interesting to see if, with such a scheme, continuity in the neuromodulatory signal (with respect to the task) would be preserved.

Third, it would be a logical progression to tackle other benchmarks to see if the observations made here hold true. More generally, analysing the neuromodulatory signal to a greater depth (and its impact on activation functions) with respect to different more complex tasks would be worthwhile. An interesting point raised in this work is that, for some tasks, neurons have been shown to have a scaling factor of zero, making their activation constant with respect to the input. Generally, any neuron that has a constant output can be pruned if the corresponding offset is added to its connected neurons. This has two interesting implications. First, some neurons have a scale factor of zero for all of the tasks and thus, by using this information, one could prune the main network without losing performance. Second, neurons having a zero-scale factor for some tasks essentially leads to only a sub-network being used for the given task. It would be interesting to discover if very different sub-networks would emerge when an NMN is trained on tasks with fewer similarities than those used in this work.

Finally, we should emphasize that even if the results obtained by our NMN are good and also rather robust with respect to a large choice of parameters, further research is certainly still needed to better characterise the NMN performances.

## Supporting information

S1 File
