# Supplementary material for: Introducing neuromodulation in deep neural networks to learn adaptive behaviours
Source: PLoS One. 2020 Jan 27;15(1):e0227922. doi: 10.1371/journal.pone.0227922 (PMC6984695; doi:10.1371/journal.pone.0227922)
Supplement: S1 File [file pone.0227922.s001.pdf]

Supporting information

1 Detailed description of benchmark 2 and 3

Before defining the three benchmark problems, let us remind that for each benchmark, the MDPs that belong to the support of $\mathcal{D}$, which generates the different tasks, have transition probabilities and reward functions that differ only according to the value of a scalar α. Drawing an MDP according to $\mathcal{D}$ will amount for all the benchmark problems to draw a value of α according to a probability distribution $P_\alpha(\cdot)$ and to determine the transition function and the reward function that correspond to this value. Let us also denote by $\mathcal{X}$ and $\mathcal{A}$ the state and action spaces respectively.

1.1 Benchmark 2

State space and action space:

$$\mathcal{X} = [-3.0, 3.0]^2$$

$$\mathcal{A} = \mathbb{R}$$

Probability distribution of α:

$$\boldsymbol{\alpha[i]} \sim \mathbb{U}[-1.0, 1.0],\ \forall i \in [1, 2]$$

$$\boldsymbol{\alpha[3]} \sim \mathbb{U}[-\pi, \pi[$$

where $\mathbb{U}[a, b]$ stands for a uniform distribution between a and b.

Initial state distribution:
The initial state x_0 is drawn through 2 auxiliary random variables that represent the x and y initial coordinates of the agent and are denoted u_0^x, u_0^y. At the beginning of an episode, those variables are drawn as follows:

$$u_0^k \sim \mathbb{U}[-1.5 * \pi, 1.5 * \pi]\ \forall k \in \{x, y\}$$

From those four auxiliary variables, we define x_0 as:

$$x_0 = [\boldsymbol{\alpha[1]} - a_0^x, \boldsymbol{\alpha[2]} - a_0^y]$$

The distribution $P_{x_0}(\cdot)$ is thus fully given by the distributions over the auxiliary variables.

Transition function:
Fist, let *target* be the set of points $(x, y) \in \mathbb{R}^2$ such that

$$(x, y) \in target \Leftrightarrow \sqrt{(x - \boldsymbol{\alpha}[\mathbf{1}])^2 + (y - \boldsymbol{\alpha}[\mathbf{2}])^2} \leq 0.4 \quad .$$

When taking action a_t in state x_t drawing the state x_{t+1} from the transition function amounts to first compute u^x_{t+1} and u^y_{t+1} according to the following procedure:

1. If $(u^x_t, u^y_t) \in target$ then $u^k_{t+1} \sim \mathbb{U}[-1.5, 1.5] \; \forall k \in \{x, y\} \quad .$
2. If the preceding condition is not met, an auxiliary variable $n_t \sim \mathbb{U}[\frac{-\pi}{4}, \frac{\pi}{4}]$ is drawn to compute u^x_{t+1} and u^y_{t+1} through the following sub-procedure:
   (a) Step one:

   $$u^x_{t+1} = u^x_t + 0.25 * (\sin(a_t) + \sin(\boldsymbol{\alpha}[\mathbf{3}] + n_t))$$
   $$u^y_{t+1} = u^x_t + 0.25 * (\cos(a_t) + \cos(\boldsymbol{\alpha}[\mathbf{3}] + n_t)) \quad .$$

   One can see that taking an action a_t moves the agent in a direction which is the vectoral sum of the intended move $\mathbf{m}_t$ of direction a_t and of a perturbation vector $\mathbf{p}_t$ of direction $\alpha + n_t$ sampled through the distribution over n_t.

   (b) Step two: In the case where the coordinates computed by step one lay outside $S[-2; 2]^2$, they are corrected so as to model the fact that when the agent reaches an edge of the 2D space, it is moved to the opposite edge from which it continues its move. More specifically, $\forall k \in \{x, y\}$:

   $$u^k_{t+1} \leftarrow \begin{cases} u^k_{t+1} - 4 & \text{if } u^k_{t+1} > 2 \\ u^k_{t+1} + 4 & \text{if } u^k_{t+1} < -2 \\ u^k_{t+1} & \text{otherwise} \quad . \end{cases}$$

Once u^x_{t+1} and u^y_{t+1} have been computed, x_{t+1} is set equal to $[\boldsymbol{\alpha}[\mathbf{1}] - u^x_{t+1}, \boldsymbol{\alpha}[\mathbf{2}] - u^y_{t+1}]$.

Reward function:
The reward function can be expressed as follows:

$$\rho(a_t, x_t, x_{t+1}) = \begin{cases} 100 & \text{if } (u^x_t, u^y_t) \in target \\ -2 & \text{otherwise} \quad . \end{cases}$$

1.2 Benchmark 3

State space and action space:

$$\mathcal{X} = [-2.5, 2.5]^4$$

$$\mathcal{A} = \mathbb{R}$$

Probability distribution of α:

$$\boldsymbol{\alpha[i]} \sim \mathbb{U}[-1.0, 1.0],\ \forall i \in [1, 2, 3, 4]$$

$$\boldsymbol{\alpha[5]} \sim \mathbb{U}\{-1, 1\}$$

Note that $\boldsymbol{\alpha}[1, 2, 3, 4]$ define the 2-D positions of two targets. For clarity, we will refer to these values respectively by α^{x_1},α^{y_1},α^{x_2} and α^{y_2}.

Initial state distribution:
The initial state x_0 is drawn through two auxiliary random variables that represent the x and y initial coordinates of the agent and are denoted u_0^x, u_0^y. At the beginning of an episode, those variables are drawn as follows:

$$u_0^k \sim \mathbb{U}[-1.5, 1.5]\ \forall k \in \{x, y\} \quad .$$

From those six auxiliary variables, we define x_0 as:

$$x_0 = [\alpha^{x_1} - u_0^x, \alpha^{y_1} - u_0^y, \alpha^{x_2} - u_0^x, \alpha^{y_2} - u_0^y] \quad .$$

Transition function:
For all $i \in \{1, 2\}$ let $target_i$ be the set of points $(x, y) \in \mathbb{R}^2$ such that

$$\sqrt{(x - \alpha^{x_i})^2 + (y - \alpha^{y_i})^2} \leq 0.4 \quad .$$

. When taking action a_t in state x_t, drawing the state x_{t+1} from the transition function amounts to first compute u_{t+1}^x and u_{t+1}^y according to the following procedure:

1. If $\exists i \in \{1, 2\} : (u_t^x, u_t^y) \in target_i$, which means that the agent is in one of the two targets, then $u_{t+1}^k \sim \mathbb{U}[-1.5, 1.5]\ \forall k \in \{x, y\}$
2. If the preceding condition is not met, u_{t+1}^x and u_{t+1}^y are computed by the following sub-procedure:
   (a) Step one:
   $$u_{t+1}^x = u_t^x + \sin(a_t * \pi) * 0.25$$
   $$u_{t+1}^y = u_t^y + \cos(a_t * \pi) * 0.25 \quad .$$
   This step moves the agent in the direction it has chosen.
   (b) Step two: In the case where the coordinates computed by step one lay outside $[-2; 2]^2$, they are corrected so as to model the fact that when the agent reaches an edge of the 2D space, it is moved to the opposite edge from which it continues its move. More specifically, $\forall k \in \{x, y\}$:
   $$u_{t+1}^k \leftarrow \begin{cases} u_{t+1}^k - 4.0 & \text{if } u_{t+1}^k > 2 \\ u_{t+1}^k + 4.0 & \text{if } u_{t+1}^k < -2 \\ u_{t+1}^k & \text{otherwise} \quad . \end{cases}$$

Once u_{t+1}^x and u_{t+1}^y have been computed, x_{t+1} is set equal to $[\alpha^{x_1} - u_{t+1}^x, \alpha^{y_1} - u_{t+1}^y, \alpha^{x_2} - u_{t+1}^x, \alpha^{y_2} - u_{t+1}^y]$.

Reward function:
In the case where (u_t^x, u_t^y) either belongs to only $target_1$, only $target_2$ or none of them, the reward function can be expressed as follows:

$$\rho(a_t, x_t, x_{t+1}) = \begin{cases} 100 * \boldsymbol{\alpha}[5] & \text{if } (u_t^x, u_t^y) \in target_1 \wedge (u_t^x, u_t^y) \notin target_2 \\ -50 * \boldsymbol{\alpha}[5] & \text{if } (u_t^x, u_t^y) \in target_2 \wedge (u_t^x, u_t^y) \notin target_1 \\ 0 & \text{if } (u_t^x, u_t^y) \notin target_1 \wedge (u_t^x, u_t^y) \notin target_2 \end{cases} .$$

In the case where (u_t^x, u_t^y) belongs to both $target_1$ and $target_2$, that is $(u_t^x, u_t^y) \in target_1 \wedge (u_t^x, u_t^y) \in target_2$, the reward function can be expressed as follows:

$$\rho(a_t, x_t, x_{t+1}) = \begin{cases} 100 * \boldsymbol{\alpha}[5] & \text{if } \sqrt{(u_t^x - p^{x_1})^2 + (u_t^y - p^{y_1})^2} \leq \sqrt{(u_t^x - p^{x_2})^2 + (u_t^y - p^{y_2})^2} \\ -50 * \boldsymbol{\alpha}[5] & \text{otherwise} \end{cases} .$$

That is, we consider that the agent belongs to the target to which it is closer to the centre.

2 Advantage actor-critic with generalized advantage estimation

In our meta-RL setting, both the actor and the critic are parametric functions that are defined on the trajectories' histories. With $\theta \in \Theta$ and $\psi \in \Psi$ the parameters of the actor and critic (Θ and Ψ are the actor and critic parameters spaces), respectively, we define π_θ and c_ψ as the policy and critic functions. Let π_{θ_k} and c_{ψ_k} be the models for the policy and the critic after k updates of the parameters θ and ψ, respectively. To update from θ_k to θ_{k+1} and ψ_k to ψ_{k+1}, the actor-critic algorithm uses the policy π_{θ_k} to select actions during B MDPs drawn sequentially from $\mathcal{D}$, where $B \in \mathbb{N}_0$ is a parameter of the actor-critic approach. This interaction between the actor-critic algorithm and the meta-RL problem is presented in a tabular version in Algorithm 1 (2.1).

Using the $L \in \mathbb{N}_0$ first elements of each trajectory generated from the interaction with the B MDPs and the values of θ_k and ψ_k, the algorithm computes θ_{k+1} and ψ_{k+1}. To this end, the algorithm exploits the set $[h_{B*k,L}, \ldots, h_{B*(k+1)-1,L}]$, which we denote as H_k. Note that we use a replay buffer for updating ψ, thus for this update we also use several previous sets H_{k-1}, H_{k-2}, etc... A tabular version of the algorithm that details how MDPs are drawn and played, as well as how the set H_k is built, is presented in Algorithm 2 of Appendix 2.1. Let $R_{\mathcal{M}}^{\pi_\theta}$ denote the sum of discounted rewards obtained when playing policy π_θ on task $\mathcal{M}$. That is,

$$R_{\mathcal{M}}^{\pi_\theta} = \lim_{T \to \infty} \sum_{t=0}^{T} \gamma^t r_t$$

where r_t are the rewards gathered at each time-step. To have a properly performing actor-critic algorithm, the value chosen for L has to be chosen sufficiently large to

produce an accurate estimation of the returns $R^{\pi_{\theta_k}}_{\mathcal{M}_i}$ $\forall i \in [B * k, \ldots, B * (k+1) - 1]$ obtained by the policy π_{θ_k}.

When used in a classical RL setting, an AC algorithm should interact with its environment to find the value of θ that leads to high values of the expected return given a probability distribution over the initial states. This expected return is written as:

$$\underset{\substack{x_0 \sim P_{x_0}(\cdot) \\ a_t \sim \pi_\theta \forall t}}{\mathbb{E}} R^{\pi_\theta}_{\mathcal{M}} \tag{1}$$

where $\mathcal{M}$ denotes the Markov Decision Process with which the AC algorithm interacts. When working well, actor critic algorithms produce a sequence of policies π_{θ_1}, π_{θ_2}, π_{θ_3}, ... whose expected returns increase as the iterative process evolves and eventually reaches values close to those obtained by $\pi_{\theta^*_{\mathcal{M}}}$ with $\theta^*_{\mathcal{M}} = \arg\max_{\theta \in \Theta} \underset{\substack{x_0 \sim P_{x_0}(\cdot) \\ a_t \sim \pi_\theta \forall t}}{\mathbb{E}} R^{\pi_\theta}_{\mathcal{M}}$, which, if π^θ is flexible enough, are themselves close to those obtained by an optimal policy $\pi^*_{\mathcal{M}}$ defined as:

$$\pi^*_{\mathcal{M}} \in \arg\max_{\pi \in \Pi} \underset{\substack{x_0 \sim P_{x_0}(\cdot) \\ a_t \sim \pi_\theta \forall t}}{\mathbb{E}} R^{\pi}_{\mathcal{M}} \tag{2}$$

where Π is the set of all admissible policies.

Let $h_t = \{x_0, a_0, r_0, \ldots, x_t\}$ be a trajectory generated by policy π_θ on $\mathcal{M}$ and let $J^{\pi_\theta}_{\mathcal{M}}(h_t)$ be the expected sum of discounted rewards that can be obtained while starting from h_t and playing the policy π_θ in this environment, that is:

$$J^{\pi_\theta}_{\mathcal{M}}(h_t) = \sum_{j=t}^{\infty} \gamma^{j-t} \rho_{\mathcal{M}}(x_j, a_j \sim \pi_\theta(h_j), x_{j+1}) \tag{3}$$

where $\rho_{\mathcal{M}}(x_j, a_j, x_{j+1})$ is the reward function of task $\mathcal{M}$. In a classical RL setting, and again for an efficient AC algorithm, the value of the critic for h_t, $c_\psi(h_t)$, also converges to $J^{\pi_{\theta^*_{\mathcal{M}}}}_{\mathcal{M}}(h_t)$. We also note that in such a setting, the critic is updated at iteration $k+1$ in a direction that provides a better approximation of $J^{\pi_{\theta_k}}_{\mathcal{M}}(\cdot)$. Now, let us go back to our meta-RL problem and let V^π denote the expected sum of returns that policy π can obtain on this problem:

$$V^\pi = \underset{\substack{x_0 \sim P_{x_0}(\cdot) \\ a_t \sim \pi_\theta \forall t \\ \mathcal{M} \sim \mathcal{D}}}{\mathbb{E}} \quad . \tag{4}$$

Let $\theta^* \in \arg\max_{\theta \in \Theta} V^{\pi_\theta}$. When interacting with our meta-RL problem, a performant AC algorithm should, in principle, converge towards a policy $\pi_{\hat{\theta}^*}$, leading to a value of $V^{\pi_{\hat{\theta}^*}}$ close to $V^{\pi^*_\theta}$ that is itself close to $\max_{\pi \in \Pi} V^\pi$. A policy π^* such that $\pi^* \in \arg\max_{\pi \in \Pi} V^\pi$ is called a Bayes optimal policy in a Bayesian RL setting where the distribution $\mathcal{D}$ is assumed to be known. If we are working with policies that are, indeed, able to quickly adapt to the environment, we may also expect that the policy $\pi_{\hat{\theta}^*}$ learned by the algorithm is such that, when applied on an $\mathcal{M}$ belonging to the support of $\mathcal{D}$, it leads to a value of $J^{\pi_{\hat{\theta}^*}}_{\mathcal{M}}(h_t)$ close to $\max_{\pi \in \Pi} J^\pi_{\mathcal{M}}(h_t)$ as t increases. In other words, once the agent has gathered enough information to adapt to the current MDP, it should start behaving (almost) optimally. This is the essence of meta-RL.

We may also expect that, in such case, the value of the critic for h_t when the budget is exhausted closely estimates the expected value of the future discounted rewards that

can be obtained when using policy $\pi^{\hat{\theta}^*}$ and after having already observed a trajectory h_t. Therefore, we may also expect that once the episode budget is exhausted, $c_\psi(h_t)$:

1. will be close to $\underset{\mathcal{M}\sim\mathcal{D}}{\mathbb{E}} J^{\pi_{\hat{\theta}^*}}_{\mathcal{M}}(h_t) \simeq \underset{\mathcal{M}\sim\mathcal{D}}{\mathbb{E}} \max_{\pi\in\Pi} J^{\pi}_{\mathcal{M}}(h_t)$ if $h_t = \{x_0\}$;
2. will, as t increases, tend to get closer to $\max_{\pi\in\Pi} J^{\pi}_{\mathcal{M}}(h_t) \simeq J^{\pi_{\hat{\theta}^*}}_{\mathcal{M}}(h_t)$ where $\mathcal{M}$ can be any environment belonging to the support of $\mathcal{D}$ used to generate h_t.

Existing actor-critic algorithms mainly differ from each other by the way the actor and critic are updated. While in early actor-critic algorithms the critic was directly used to compute the direction of update for the actor's parameters (see for example the REINFORCE policy updates [1]), now it is more common to use an advantage function. This function represents the advantage in terms of return of selecting specific actions given a trajectory history (or simply a state when AC algorithms are used in a standard setting) over selecting them following the policy used to generate the trajectories. Here, we use generalised advantage estimations (GAE), as introduced in [2]. More recently, it has been shown that avoiding too large policy changes between updates can greatly improve learning ([3], [4]). Therefore, while in classical AC algorithms the function used to update the actor aims at representing directly the gradient of the actor's return with respect to its parameters, we rather update the actor's parameters θ by minimising a loss function that represents a surrogate objective. We have selected as surrogate function one that is similar to the one introduced in [4] with an additional loss term that proved to improve (albeit slightly) the performances of PPO in all cases.

As our actor and critic are modelled by differentiable functions, they are both updated through gradient descent. We now proceed to explain the losses use to compute the gradient for both the actor and the critic.

Actor update First, we define the temporal error difference term for any two consecutive time-steps of any trajectory:

$$TD_i = r_i + \gamma * c_{\psi_k}(h_{i+1}) - c_{\psi_k}(h_i),\ \forall i \in [0, \dots, L]$$

where ψ_k denotes the critic's parameters for playing the given trajectory. This temporal difference term represents, in some sense, the (immediate) advantage obtained, after having played action a_j over what was expected by the critic. If $c_{\psi_k}(\cdot)$ was the true estimate of $J^{\pi_{\theta_k}}_{\mathcal{M}}(\cdot)$ and if the policy played was π_{θ_k}, the expected value of these temporal differences would be equal to zero. We now define the GAE's terms that will be used later in our loss functions:

$$GAE^i_j = \sum_{t=j}^{L} (\gamma * \lambda)^{t-j} * TD^j_i,\ \forall j \in [1, \dots, E], i \in [0, \dots, L'] \quad (5)$$

where $\lambda \in [0, 1]$ is a discount factor used for computing GAEs, TD^j_i is the value of TD_i for trajectory j, and where L' is another hyper-parameter of the algorithm, chosen in combination with L in order to have a value of $GAE_{i,j}$ that accurately approximates

$\sum_{t=j}^{\infty}(\gamma * \lambda)^{k-j} * TD_i^j \ \forall i, j$. Note that the value chosen for L' also has to be sufficiently large to provide the loss function with a sufficient number of GAE terms. These GAE terms, introduced in [2], represent the exponential average of the discounted future advantages observed. Thanks to the fact that GAE terms can catch the accumulated advantages of a sequence of actions rather than of a single action, as it is the case with the temporal difference terms, they can better represent the advantage of the new policy played by the AC algorithm over the old one (in terms of future discounted rewards).

In the loss function, we will actually not use the advantage terms as defined by Equation 5, but normalised versions in order to have advantages that remain in a similar range regardless of rewards magnitude. Thanks to this normalisation, the policy learning rate does not have to be tuned according to the loss magnitude. However, this normalisation does not mask actions that have led to higher or lower returns than average. We normalize as follows $\forall k \in [1, \ldots, E]$ with E the number of actor and critic updates that have been carried:

$$\mu_{gae} = \sum_{j=0}^{B-1}[\sum_{i=0}^{L'-1} GAE_i^{B*k+j}]$$

$$\sigma_{gae} = \sqrt{\sum_{j=0}^{B-1}[\sum_{i=0}^{L'-1}(\mu_{gae} - GAE_i^{B*k+j})^2]}$$

$$GAE_i^j = \frac{GAE_i^{B*k+j} - \mu_{gae}}{\sigma_{gae}} \ \forall (j,i) \in ([0, \ldots, B-1] * [0, \ldots, L'-1])$$

where $\sum$ is the symbol we use to represent the average sum operator (i.e. $\sum_{x=1}^{m} f(x) = \sum_{x=1}^{m} \frac{f(x)}{m}$). To define the loss functions used to compute θ_{k+1} and ψ_{k+1}, only the GAE terms corresponding to time-steps $[0, \ldots, L']$ of episodes $[B * k, \ldots, B * (k+1) - 1]$ are computed. A tabular version of the algorithm used to compute these terms is given in Algorithm 3 of Appendix 2.1 [1].

Once advantages have been computed, the values of θ_{k+1} are computed using updates that are strongly related to PPO updates with a Kullback Leibler (KL) divergence implementation [4]. The loss used in PPO updates is composed of two terms: a classic policy gradient term and a penalisation term. Let us now present a standard policy gradient loss, note that from now on, we will refer to the value of a_t, x_t and h_t at episode i by a_t^i, x_t^i and h_t^i respectively.

$$\mathcal{L}_{vanilla}(\theta) = - \sum_{(i,t) \in \mathcal{B}_k} \frac{\pi_\theta(a_t^i | h_t^i)}{\pi_{\theta_k}(a_t^i | h_t^i)} * GAE_t^i \quad . \tag{6}$$

[1] Although not explicitly written in the text for clarity, we use a normalisation technique when computing discounted sums for the AC algorithm update. In fact, when carrying an update of the AC algorithm, if rewards appear in discounted sums, they are multiplied by $(1-\gamma)$. This has for effect that the discounted sum values remain of the same magnitude regardless of γ. The implications of this normalization are two-fold. (i) The critic does not directly approximate $J_{\mathcal{M}}^{\pi}(\cdot)$ but rather $(1-\gamma) * J_{\mathcal{M}}^{\pi}(\cdot)$. (ii) Second, for the temporal differences to remain coherent with this normalisation, r_i must also be multiplied by $(1-\gamma)$ when computing TD_i. Those two small changes are included in Algorithm 3.

where $\mathcal{B}_k$ is the set of all pairs (i, t) for which $i, t \in ([B*k, \ldots, B*(k+1)-1]*[0, \ldots, L'])$, that is, the set containing the first L' time-steps of the B trajectories played for iteration k of the actor-critic algorithm.

One can easily become intuitive about Equation 6 as, given an history h_t^i, minimising this loss function tends to increase the probability of the policy taking actions leading to positive advantages (i.e. $GAE_t^i > 0$) and decreases its probability to take actions leading to negative advantages (i.e. $GAE_t^i < 0$). It has been found that to obtain good performances with this above-written loss function, it was important to have a policy that does not change too rapidly from one iteration to the other. Before explaining how this can be achieved, let us first give an explanation on why it may be important to have slow updates of the policy. Let us go back to the loss function given by Equation 6. Minimising this loss function will give a value for θ_{k+1} that will lead to higher probabilities of selecting actions corresponding to high values of the advantages GAE_t^i. A potential problem is that these advantages are not really related to the advantages of the would-be new policy $\pi_{\theta_{k+1}}$ over π_{θ_k} but are instead related to the advantages of policy π_{θ_k} over $\pi_{\theta_{k-1}}$. Indeed, the advantages GAE_t^i are computed using the value function c_{ψ_k}, whose parameters have been updated from ψ_{k-1} in order to better approximate the sum of discounted rewards obtained during the episodes $[B*(k-1), \ldots, B*k-1]$. It clearly appears that ψ_k has, in fact, been updated to approximate discounted rewards obtained through the policy $\pi_{\theta_{k-1}}$ (used to play episodes for update $k-1$). A solution to this problem is to constraint the minimisation to reach a policy $\pi_{\theta_{k+1}}$ that does not stand too far from π_{θ_k}. We may reasonably suppose that the advantage function used in (6) still correctly reflects the real advantage function of $\pi_{\theta_{k+1}}$ over π_{θ_k}. To achieve this, we add a penalisation term $\mathcal{P}(\theta)$ to the loss function. In the PPO approach, the penalisation term is $\mathcal{P}_{ppo}(\theta) = \beta_k * d(\theta)$, where:

i) β_k is an adaptive weight

ii) $d(\theta) = \sum_{[i,t]\in\mathcal{B}_k} [KL(\pi_{\theta_k(.|h_{i,t})}, \pi_{\theta(.|h_{i,t})})]$, where KL is the Kullback-Leibler divergence, detailed later on. This term penalises policies that are too different from π_{θ_k}.

We note that the β_k dynamical updates use a hyper-parameter $d_{targ} \in \mathbb{N}_0$ called the divergence target. The update is done through the following procedure (note that, unlike updates of β proposed in [4], we constrain β to remain in the range $[\beta_{min}, \beta_{max}]$; we explain later why):

$$\beta_{k+1} = \begin{cases} \max(\beta_{min}, \frac{\beta_k}{1.5}) & \text{if } d(\theta) < \frac{d_{targ}}{2.0} \\ \min(\beta_{max}, \beta_k * 1.5) & \text{if } d(\theta) > d_{targ} * 2 \\ \beta_k & \text{otherwise} \quad . \end{cases} \tag{7}$$

With this update strategy, the penalisation term will tend to evolve in a way such that the KL divergence between two successive policies does not tend to go beyond d_{targ} without having to add an explicit constraint on d, as was the case in Trust Region Policy Optimization (TRPO) updates [3], which is more cumbersome to implement.

As suggested in [5], adding another penalisation term (squared hinge loss) to $\mathcal{P}_{PPO}$ to further penalise the KL divergence, in cases where it surpasses $2 * d_{targ}$, improved algorithm performance. The final expression of the penalisation term is:

$$\mathcal{P}(\theta) = \beta_k * d(\theta) + \delta * \max(0, d(\theta) - 2 * d_{targ})^2$$

where δ is a hyper-parameter that weights the third loss term. The loss function $\mathcal{L}_{policy}$ that we minimise as a surrogate objective becomes:

$$\mathcal{L}_{policy}(\theta) = \mathcal{L}_{vanilla}(\theta) + \mathcal{P}(\theta) \tag{8}$$

We now detail how to compute the KL divergence. First, let us stress that we have chosen to work with multi-variate Gaussian policies for the actor. This choice is particularly well suited for MDPs with continuous action spaces. The approximation architecture of the actor will therefore not directly output an action, but the means and standard deviations of an m-dimensional multi-variate Gaussian from which the actor's policy can be defined in a straightforward way. For each dimension, we bound the multi-variate Gaussian to the support, $\mathcal{U}$, by playing the action that is clipped to the bounds of $\mathcal{U}$ whenever the multi-variate Gaussian is sampled outside of $\mathcal{U}$. In the remaining of this paper, we will sometimes abusively use the terms "output of the actor at time t of episode i" to refer to the means vector $\mu_{i,t}^{\theta_k}$ and the standard deviations vector $\sigma_{i,t}^{\theta_k}$ that the actor uses to define its probabilistic policy at time-step t of episode i. Note that we have chosen to work with a diagonal covariance matrix for the multi-variate Gaussian distribution. Its diagonal elements correspond to those of the vector $\sigma_{i,t}^{\theta_k}$. We can then compute the KL divergence in each pair $[i, t]$ following the well-established formula:

$$KL(\pi_{\theta_k}(\cdot|h_t^i), \pi_\theta(\cdot|h_t^i)) = \frac{1}{2}\{tr(\Sigma_{\theta,i,t}^{-1}\Sigma_{\theta_k,i,t}) + (\mu_{i,t}^{\theta} - \mu_{i,t}^{\theta_k})^T \Sigma_{\theta,i,t}^{-1}(\mu_{i,t}^{\theta} - \mu_{i,t}^{\theta_k}) - k + \ln(\frac{|\Sigma_{\theta,i,t}|}{|\Sigma_{\theta_k,i,t}|})\} \tag{9}$$

where $\Sigma_{\theta_k,i,t}, \Sigma_{\theta,i,t}$ are the diagonal covariance matrices of the two multi-variate Gaussian distributions $\pi_{\theta_k}(\cdot|h_t^i), \pi_\theta(\cdot|h_t^i)$ that can be derived from $\sigma_{i,t}^{\theta_k}$ and $\sigma_{i,t}^{\theta}$. The loss function $\mathcal{L}_{vanilla}$ can be expressed as a function of $\Sigma_{\theta_k,i,t}$, $\Sigma_{\theta,i,t}$, $\mu_{i,t}^{\theta}$ and $\mu_{i,t}^{\theta_k}$ when working with a multi-variate Gaussian. To this end, we use the log-likelihood function $\ln(\pi_\theta(a_{i,t}|h_t^i))$, which gives the log-likelihood of having taken action a_t^i given a trajectory history h_t^i. In the case of a multi-variate Gaussian, $\ln(\pi_\theta(a_t^i|h_t^i))$ is defined as:

$$\ln(\pi_\theta(a_t^i|h_t^i)) = -\frac{1}{2}(\ln(|\Sigma_{\theta,i,t}|) + (a_t^i - \mu_{i,t}^{\theta})^T * \Sigma_{\theta,i,t}^{-1} * (a_t^i - \mu_{i,t}^{\theta}) + m * \ln(2 * \pi)) \tag{10}$$

where m is the dimension of the action space and where $|\Sigma_{\theta,i,t}|$ represents the determinant of the matrix. From this definition, one can rewrite $\mathcal{L}_{vanilla}$ as:

$$\mathcal{L}_{vanilla} = -\sum_{[i,t]\in\mathcal{B}_k} e^{\ln(\pi_\theta(a_t^i|h_t^i)) - \ln(\pi_{\theta_k}(a_t^i|h_t^i))} * GAE_t^i \quad . \tag{11}$$

By merging equation (11), (10) and equation (8), one gets a loss $\mathcal{L}_{policy}$ that depends only on $\Sigma_{\theta_k,i,t}$, $\Sigma_{\theta,i,t}$, $\mu_{i,t}^{\theta}$ and $\mu_{i,t}^{\theta_k}$.

Critic update The critic is updated at iteration k in a way to better approximate the expected return obtained when following the policy π_{θ_k}, starting from a given trajectory

history. To this end, we use a mean-square error loss as a surrogate objective for optimizing ψ. First, we define $\hat{R}^i_j = \sum_{k=j}^{L} \gamma^{k-j} * r^i_j \; \forall i, j \in [B * k, \ldots, B * (k+1) - 1], [0, \ldots, L]$. From the definition of $\hat{R}^i_j$ we express the loss as:

$$\mathcal{L}_{critic}(\psi) = \sum_{(i,t) \in \mathcal{B}_{k-CRB}} [(c_\psi(h^i_t) - \hat{R}^i_t)^2] \tag{12}$$

where (i) $crb \in \mathbb{N}_0$ is a hyper-parameter; (ii) $\mathcal{B}_{k-crb}$ is the set of all pairs (i, t) for which $i, j \in ([B*(k-crb), \ldots, B*(k+1)-1]*[0, \ldots, L'])$. The set $\mathcal{B}_{k-crb}$ used in (12) contains all the pairs from the current trajectory batch and from the crb previous trajectory batches. We call this a replay buffer whose length is controlled by crb which stands for "**c**ritic**r**eplay**b**uffer". Minimising $\mathcal{L}_{critic}$ does not lead to updates such that c_ψ directly approximates the average expected return of the policy π_{θ_k}. Rather, the updates are such that c_ψ directly approximates the average expected return obtained by the last $crb + 1$ policies played. We found out that using a replay buffer for the critic smoothed the critic's updates and improved algorithm performances.

Note that the loss (12) is only computed on the $L' << L$ first time-steps of each episode, as was the case for the actor. The reason behind this choice is simple. The value function c_{ψ_k} should approximate $R^i_j = \sum_{t=j}^{+\infty} \gamma^{t-j} * r^i_t$ for every h^i_j, where R^i_j the infinite sum of discounted rewards that are attainable when "starting" from h^i_j. However, this approximation can become less accurate when j becomes close to L since we can only guarantee $\hat{R}^i_j$ to stand in the interval: $[R^i_j - \frac{\gamma^{L-j}}{1-\gamma} R_{max}, R^i_j - \frac{\gamma^{L-j}}{1-\gamma} R_{min}]$. Hence this choice of L'.

Gradients computation and update The full procedure is available as Tabular versions in 2.1. As a summary, we note that both the actor and critic are updated using the Adam procedure ([6]) and back-propagation through time ([7]). The main difference between both updates is that the actor is updated following a full-batch gradient descent paradigm, while the critic is updated following a mini-batch gradient descent paradigm.

2.1 Tabular version

Algorithm 1 Advantage actor-critic with generalised advantage estimate for solving the meta-RL problem

Run($\mathcal{D}$, E, $\mathbf{hyperparameters_0}$)
Inputs:

[1] $\mathcal{D}$: The distribution over MDPs.

[2] E : The total episodes budget.

[3] $hyperparameters_0$: The set of hyper-parameters that contains the following elements:

- B : Number of episodes played between updates.
- P_{θ_0} and P_{ψ_0} : The distributions for initialising actor and critic's parameters. Those distributions are intrinsically tied to the models used as function approximators.
- $\lambda \in [0, 1]$: The discount factor for computing GAE.
- L : Number of time steps played per episode.
- L' : Number of time steps per episode used to compute gradients.
- e_a : The number of epochs per actor update.
- e_c : The number of epochs per critic update.
- η : The squared hinge loss weight.
- d_{targ} : The KL divergence target.
- d_{thresh} : The threshold used for early stopping.
- β_{min} and β_{max} : The minimum and maximum β values.
- β_0 : The initial value of β_k for penalising the KL divergence.
- a_{lr_0} : The initial value of the policy learning rate a_{lr_k}.
- c_{v_0}, c_{z_0}, a_{v_0} and a_{z_0} : The initial value for the ADAM optimiser moments c_{v_k}, c_{z_k}, a_{v_k} and a_{z_k}.
- ϵ, ω_1, ω_2 : The three ADAM optimiser hyper-parameters.
- c_{lr} : The critic learning rate.
- cmb : The mini-batch size used for computing the critic's gradient.
- crb : The number of previous trajectory batches used in the replay buffer for the critic.

We note that some of the hyper-parameters are adaptive. These are β_k, a_{lr_k}, c_{v_k}, c_{z_k}, a_{v_k} and a_{z_k}. Thus the hyper-parameter vector may have to change in between iterations. For this reason we introduce the notation hp_k which represents the hyper-parameter vector with the values of the adaptive parameters at iteration k.

$hp_0 \leftarrow hyperparameter_0$
$k \leftarrow 0$
$\theta_0 \sim P_{\theta_0}(.)$ ▷ Random initialisation
$\psi_0 \sim P_{\psi_0}(.)$ ▷ Random initialisation
while $B * k < E$ **do**
    $H_k =$ **run episodes**$(k, \theta_k, \mathcal{D}, hp_k)$
    $\theta_{k+1}, \psi_{k+1} =$ **update ac**$(H_{\max(0,k-CRB)}, \ldots, H_k, \theta_k, \psi_k, hp_k)$
    $k \leftarrow k + 1$

Algorithm 2 Kth run of B episodes

run episodes(k, θ_k, $\mathcal{D}$, hp_k)

Inputs:

[1] θ_k : The parameters of the policy at iteration k.

[2] $\mathcal{D}$: The distribution from which the MDPs are sampled.

[3] hp_k : In this procedure, we use as hyper-parameters:

- B : The number of episodes to be played.
- L : The number of time steps played by episode.

Output:

[1] H^k : The set of B trajectories $[h_L^{B*k}, h_{B*(k+1),L}, \ldots, h_L^{B*(k+1)-1}]$ played during this procedure.

$i \leftarrow B * k$

while $i < B * k + B$ **do**

  $t \leftarrow 0$

  $\mathcal{M} \sim \mathcal{D}$

  $x_t^i \sim P_{x_0}(.)$

  $h_t^i = [x_t^i]$

  while $t <= L$ **do**

    $a_t^i \sim \pi_{\theta_k}(h_t^i)$

    $x_{t+1}^i \sim P^{\mathcal{M}}(x_{t+1}^i | x_t^i, a_t^i)$ ▷ The right-side refers to $P(x_{t+1}^i | x_t^i, a_t^i)$ of current task $\mathcal{M}$.

    $r_t^i = \rho^{\mathcal{M}}(x_t^i, a_t^i, x_{t+1}^i)$ ▷ The right-side refers to $\rho(x_{t+1}, x_t, a_t)$ of $\mathcal{M}$.

    $h_t^i = [x_0^i, a_0^i, r_0^i, \ldots, x_t^i]$

    $t \leftarrow t + 1$

  $i \leftarrow i + 1$

Return $H_k = [h_L^{B*k}, \ldots, h_L^{B*(k+1)-1}]$

Algorithm 3 Kth update of the actor critic model

update ac($H^{k-crb}, \ldots, H^k$, θ_k, ψ_k, hp_k)
Inputs:
   [1] $H^{k-crb}, \ldots, H^k$: The $crb+1$ last sets of B trajectories of length L.
   [2] θ_k and ψ_k : The parameters of the actor and critic after k updates.
   [3] hp_k : In this procedure, we use as hyper-parameter:
      •$\lambda \in [0, \ldots, 1]$: The discount factor for computing GAE.
      •a_{lr_k} : The current policy learing rate.
      •β_k : The current KL divergence penalisation.
Output:
   [1] θ_{k+1}, ψ_{k+1} : The updated actor and critic parameters.
$i \leftarrow B * k$
$\mathbf{D} \leftarrow \emptyset$
while $i < B * k + B$ **do**
   $D^i_j = \sum_{t=j}^{L} \gamma^{t-j} * r_j * (1-\gamma),\ \forall j \in [0, \ldots, L-1]$
   $\mathbf{D} \leftarrow \mathbf{D} \cup D^i_j$
   $TD_j = (1-\gamma) * r_j - c_{\psi_k}(h_j) + c_{\psi_k}(h_{j+1}), j \in [0, \ldots, L-1]$
   $GAE^i_j = \sum_{t=j}^{L} (\gamma * \lambda)^{t-j} * TD_j,\ \forall j \in [0, \ldots, L-1]$
$\mu = \sum_{i=B*k}^{B*(k+1)-1} \sum_{j=0}^{L-1} GAE^i_j$
$\sigma = \sqrt{\sum_{i=B*k}^{B*(k+1)-1} \sum_{j=0}^{L-1} (\mu - GAE^i_j)^2}$
$GAE^i_j \leftarrow \frac{GAE^i_j - \mu}{\sigma}\ \forall i \in [B*k, \ldots, B*(k+1)-1], j \in [0, \ldots, L-1]$
$\mathbf{A} = [GAE^{B*k+i}_j,\ \forall (i,j) \in ([0, \ldots, B-1] * [0, \ldots, L])]$
θ_{k+1} = **update policy parameters**(H^k, $\mathbf{A}$, θ_k, hp_k)
ψ_{k+1}= **update critic parameters**($H^{k-crb}, \ldots, H^k$, $\mathbf{D}$, ψ_k, hp_k)
Return θ_{k+1}, ψ_{k+1}

Algorithm 4 Update from θ_k to θ_{k+1}

update policy parameters(H_k, $\mathbf{A}$,θ_k,hp_k)
Inputs:

[1] H^k : The set of B trajectories of length L.

[2] θ_k : The actor's parameters.

[3] ϵ, ω_1 and ω_2 : The three ADAM optimizer hyper-parameters.

[4] hp_k : In this procedure, we use as hyper-parameters:

•e_{actor} : The number of epochs per actor update.
•η : The squared hinge loss weight.
•d_{targ} : The KL divergence target.
•d_{thresh} : The threshold used for early stopping.
•L' : The number of time-steps per trajectory used for computing gradients.
•β_k : The KL penalisation weight.
•a_{lr_k} : The actor learning rate.
•a_{v_k} and a_{z_k} : The last ADAM moments computed at iteration $k-1$.

Output:

[1] θ_{k+1} : The updated actor parameters.

$m \leftarrow 0$
$\mathcal{B} \leftarrow [(B * k + i, j),\ \forall (i,j) \in ([0,\ldots,B-1] * [0,\ldots,L'])]$
$\theta' \leftarrow \theta_k$
$a'_v \leftarrow a_{v_k}$
$a'_z \leftarrow a_{z_k}$
while $m < AE$ **do**
  $\mathcal{L}_{vanilla} = -\sum_{(i,t)\in\mathcal{B}} \frac{\pi_\theta(a_t^i|h_t^i)}{\pi_{\theta_k}(a_t^i|h_t^i)} * GAE_t^i$
  $d = \sum_{(i,t)\in\mathcal{B}} KL(\pi_{\theta_k}(.|h_j^i), \pi_\theta(.|h_j^i))$
  $s = [\max(0, (d - 2 * d_{targ}))]^2$
  $\mathcal{L}_{policy} = \mathcal{L}_{vanilla} + \beta_k * d + \eta * s$
  $\nabla_\theta \mathcal{L}_{policy}(\theta') =$ **compute gradients**$(\mathcal{L}_{policy}, \mathcal{B}_k, \theta')$
  $a'_{lr} = a_{lr_k} * \frac{\sqrt{1-\omega_2^{k*e_{actor}+m}}}{1-\omega_1^{k*e_{actor}+m}}$
  $a'_z \leftarrow \omega_1 * a'_z + (1-\omega_1) * \nabla_\theta \mathcal{L}_{policy}(\theta')$
  $a'_v \leftarrow \omega_2 * a'_v + (1-\omega_2) * \nabla_\theta \mathcal{L}_{policy}(\theta') \odot \nabla_\theta \mathcal{L}_{policy}(\theta')$
  $\theta' \leftarrow \theta' - \frac{a'_{lr} * a'_z}{\sqrt{a'_v} + \epsilon}$
  $m \leftarrow m + 1$
  if $d > d_{threshold} * d_{targ}$ **then** ▷ Early stop
    $\theta' \leftarrow \theta_k$
    $m \leftarrow e_{actor}$
update auxiliary parameters(d, hp_k)
$a_{v_{k+1}} \leftarrow a'_v$
$a_{z_{k+1}} \leftarrow a'_z$
$\theta_{k+1} \leftarrow \theta'$
Return θ_{k+1}

Algorithm 5 Actor auxiliary parameters update

update auxiliary parameters(d,hp_k)
Inputs:
[1] d : The KL divergence between π_{θ_k} and $\pi_{\theta_{k+1}}$ empirically averaged.
[2] hp_k : In this procedure, we use as hyper-parameters:
•d_{targ} : The KL divergence target.
•β_{min} and β_{max} : The minimum and maximum β values.
•β_k : The current KL penalisation weight.
•a_{lr_k} : The current actor learning rate.
if $d > 2 * d_{targ}$ **then**
    $\beta_{k+1} \leftarrow \min(\beta_{max}, \beta_k * 1.5)$
    if $\beta_k > 0.85 * \beta_{max}$ **then**
        $a_{lr_{k+1}} \leftarrow \frac{a_{lr_k}}{1.5}$
else if $d < \frac{d_{targ}}{2}$ **then**
    $\beta_{k+1} \leftarrow \max(\beta_{min}, \frac{\beta_k}{1.5})$
    if $\beta_k < 1.15 * \beta_{min}$ **then**
        $a_{lr_{k+1}} \leftarrow a_{lr_k} * 1.5$

Algorithm 6 Update from ψ_k to ψ_{k+1}

update critic parameters$(H_{k-CRB}, \dots, H_k, \mathbf{D}, \psi_k, hp_k)$

Inputs:

[1] $H_{k-CRB}, \dots, H_k$: The $crb+1$ last sets of trajectories of length L.

[2] ψ_k : The critic's parameters.

[3] hp_k : In this procedure, we use as hyper-parameters:

- e_{critic} : The number of epochs per critic update.
- cmb : The mini-batch size used for computing the critic's gradient.
- T : A hyper-parameter of our gradient estimate.
- crb : The replay buffer size.
- c_{lr} : The critic learning rate.
- L' : The number of time-steps per trajectory used for computing gradients.
- c_{v_k} and c_{z_k} : The last ADAM moments computed at iteration $k-1$.

Output:

[1] ψ_{k+1} : The updated critic parameters.

$m \leftarrow 0$

$c'_v \leftarrow c_{v_k}$

$c'_z \leftarrow c_{z_k}$

$\mathcal{T}_{(i,t)} = [[i, t*T], \dots, [i, max((t+1)*T-1, L')]]\ \forall (i,t) \in ([B*(k-CRB), \dots, B*(k+1)-1] * [0, \dots, \lfloor \frac{L'}{T} \rfloor])$

$\mathcal{BT} = [(B*(k-crb)+i, j) \forall (i,j) \in ([0, \dots, B*(crb+1)-1] * [0, \dots, \lfloor \frac{L'}{T} \rfloor])]$

$\psi' \leftarrow \psi_k$

$e_{iter} \leftarrow e_{critic} * \lceil \frac{|\mathcal{BT}|}{cmb*T} \rceil$

$\mathcal{S} \leftarrow \emptyset$

while $m < e_{iter}$ **do**

    $p \leftarrow 0$, $\mathcal{Y} \leftarrow \emptyset$

    while $p < cmb \wedge \mathcal{BT} \setminus \mathcal{S} \neq \emptyset$ **do**

        $(i_{cur}, t_{cur}) \sim \mathcal{BT} \setminus \mathcal{S}$

        $\mathcal{S} \leftarrow \mathcal{S} \cup (i_{cur}, t_{cur})$

        $\mathcal{Y} \leftarrow \mathcal{Y} \cup \mathcal{T}_{i_{cur}, t_{cur}}$

        $p \leftarrow p+1$

    if $\mathcal{BT} \setminus \mathcal{S} = \emptyset$ **then**

        $\mathcal{S} \leftarrow \emptyset$

    $\mathcal{L}_{sur}(\psi) = \sum_{(i,t) \in \mathcal{Y}} (c_\psi(h^i_t) - D^i_t)^2$

    $\nabla_\psi \mathcal{L}_{sur}(\psi', \mathcal{Y}) =$ **compute gradients**$(\mathcal{L}_{sur}, \mathcal{Y}, \psi')$

    $c'_{lr} = c_l r * \frac{\sqrt{1-\omega_2^{k*e_{iter}+m}}}{1-\omega_1^{k*e_{iter}+m}}$

    $c'_z = \omega_1 * c'_z + (1-\omega_1) * \nabla_\psi \mathcal{L}_{sur}(\psi', \mathcal{Y})$

    $c'_v = \omega_2 * c'_v + (1-\omega_2) * \nabla_\psi \mathcal{L}_{sur}(\psi', \mathcal{Y}) \odot \nabla_\psi \mathcal{L}_{sur}(\psi', \mathcal{Y})$

    $\psi' \leftarrow \psi' - \frac{c'_{lr} * c'_z}{\sqrt{c'_v} + \epsilon}$

    $m \leftarrow m+1$

$c_{v_{k+1}} \leftarrow c'_v$

$c_{z_{k+1}} \leftarrow c'_z$

$\psi_{k+1} \leftarrow \psi'$

Return ψ_{k+1}

Algorithm 7 Gradient computing with BPTT [7]

1: **compute gradients**($\mathcal{L}(\alpha), \mathcal{Z}, \alpha'$, hp_k)

2: **Inputs**:

[1] $\mathcal{L}(\alpha)$: A loss function which is dependent on a function approximate $v(\alpha)_{\cdot,\cdot}$.

[2] $\mathcal{Z}$: The set of pairs (i, j) such that $v(\alpha)_{i,j}$ appears in $\mathcal{L}(\alpha)$. ▷ We emphasise that from the way $\mathcal{Z}$ is built (see Algorithms 4 and 6), most of the time $\mathcal{Z}$ contains x batches of T consecutive pairs. Note that very rarely, batches may have fewer than T consecutive pairs (whenever a batch contains the last pairs of an episode which does not contain a multiple of T pairs), although, the same gradient descent algorithm can still be applied.

[3] α' : The element for which the estimate gradient of $\mathcal{L}(\alpha)$ needs to be evaluated.

[4] hp_k : In this procedure, we use as hyper-parameter:

•T : The number of time-steps for which the gradient can propagate.

3: **Output**:

[1] $\nabla_\alpha \mathcal{L}(\alpha')$: The gradient estimate of the function $\mathcal{L}(\alpha)$ evaluated in α'.

▷ We refer the reader to the source code which is available on Github (`https://github.com/nvecoven/nmd_net`), which is a particular implementation of standard BPTT [7]. We note that giving a full tabular version of the algorithm here would not constitute valuable information to the reader, due to its complexity/length.

3 Architecture details

For conciseness, let us denote by f_n a hidden layer of n neurons with activation functions f, by $\rightarrow$ a connection between two fully-connected layers and by $\multimap$ () a neuromodulatory connection (as described in Section **??**).

Benchmark 1. The architectures used for this benchmark were as follows:

- RNN : $GRU_{50} \rightarrow ReLU_{20} \rightarrow ReLU_{10} \rightarrow I_1$
- NMN : $GRU_{50} \rightarrow ReLU_{20} \multimap (SReLU_{10} \rightarrow I_1)$

Benchmark 2 and 3. The architectures used for benchmark 2 and 3 were the same and as follows:

- RNN : $GRU_{100} \rightarrow GRU_{75} \rightarrow ReLU_{45} \rightarrow ReLU_{30} \rightarrow ReLU_{10} \rightarrow I_1$
- NMN : $GRU_{100} \rightarrow GRU_{75} \rightarrow ReLU_{45} \multimap (ReLU_{30} \rightarrow ReLU_{10} \rightarrow I_1)$

4 Hyper-parameter values

B	50
λ	0.98
γ	0.998
β_0	1
β_{min}	1/30
β_{max}	30
d_{targ}	0.003
a_{lr_0}	$2 * 10^{-4}$
ω_1	0.9
ω_2	0.999
ϵ	10^{-8}
e_{actor}	20
crb	2
cmb	25
c_{lr}	$6 * 10^{-3}$
T	200
e_{critic}	10
$c_{v_0}, c_{z_0}, a_{v_0}, a_{z_0}$	0
η	50

Table 1. Value of the hyper-parameters that are kept constant for every benchmark in this paper.

Table 1 provides the values of all the hyper-parameters used for training.

5 Bayes optimal policy for benchmark 1

A Bayes optimal policy is a policy that maximises the expected sum of rewards it obtains when playing an MDP drawn from a known distribution $\mathcal{D}$. That is, a Bayes optimal policy π^*_{bayes} belongs to the following set:

$$\pi^*_{bayes} \in \arg\max_{\pi \in \Pi} \underset{\substack{\mathcal{M} \sim \mathcal{D} \\ x_0 \sim P_{x_0} \\ a_\cdot \sim \pi(\cdot) \\ x_\cdot \sim P_{\mathcal{M}}(\cdot,\cdot)}}{\mathbb{E}} R^\pi_{\mathcal{M}} \quad ,$$

with $P_{\mathcal{M}}$ being the state-transition function of the MDP $\mathcal{M}$ and $R^\pi_{\mathcal{M}}$ the discounted sum of reward obtained when playing policy π on $\mathcal{M}$.

In the first benchmark, the MDPs only differ by a bias, which we denote α. Drawing an MDP according to $\mathcal{D}$ amounts to draw a value of α according to a uniform distribution of α over $[-\alpha_{max}, \alpha_{max}]$, denoted by $\mathbb{U}_\alpha$, and to determine the transition function and the reward function that correspond to this value. Therefore, we can write the previous equation as:

$$\pi^*_{bayes} \in \arg\max_{\pi \in \Pi} \underset{\substack{\alpha \sim \mathbb{U}_\alpha \\ x_0 \sim P_{x_0} \\ a_\cdot \sim \pi(\cdot) \\ x_\cdot \sim P_{\mathcal{M}(\alpha)}(\cdot,\cdot)}}{\mathbb{E}} R^\pi_{\mathcal{M}} \quad ,$$

with $\mathcal{M}(\alpha)$ being a function giving as output the MDP corresponding to α and Π the set of all possible policies.

We now prove the following theorem.

Theorem 1 *The policy that selects:*

1. *at time-step $t = 0$ the action $a_0 = x_0 + \frac{\gamma * (\alpha_{max} + 4.5)}{1+\gamma}$*
2. *at time-step $t = 1$*
   a) *if $r_0 = 10$, the action $a_1 = x_1 + a_0 - x_0$*
   b) *else if $|r_0| > \alpha_{max} - (a_0 - x_0) \quad \wedge \quad a_0 - x_0 > 0$, the action $a_1 = a_0 + r_0$*
   c) *else if $|r_0| > \alpha_{max} - (x_0 - a_0) \quad \wedge \quad a_0 - x_0 < 0$, the action $a_1 = a_0 - r_0$*
   d) *and otherwise the action $a_1 = a_0 + r_0 + 1$*
3. *for the remaining time-steps:*
   a) *if $r_0 = 10$, the action $a_t = x_t + a_0 - x_0$*
   b) *else if $r_1 = 10$, the action $a_t = x_t + a_1 - x_1$*
   c) *and otherwise the action $a_t = x_t + i_t$ where i_t is the unique element of the set $\{a_0 - x_0 + r_0; a_0 - x_0 - r_0\} \cap \{a_1 - x_1 + r_1; a_1 - x_1 - r_1\}$*

is Bayes optimal for benchmark 1.

Proof Let us denote by $\pi^*_{theorem1}$ the policy described in this theorem. To prove this theorem, we first prove that in the set of all possible policies Π there are no policy π which leads to a higher value of

$$\underset{\substack{\mathcal{M}\sim\mathcal{D}\\ x_0\sim P_{x_0}\\ a.\sim\pi(\cdot)\\ x.\sim P_{\mathcal{M}}(\cdot,\cdot)}}{\mathbb{E}} (r_0 + \gamma * r_1) \tag{13}$$

than $\pi^*_{theorem1}$. Or equivalently:

$$\underset{\substack{\mathcal{M}\sim\mathcal{D}\\ x_0\sim P_{x_0}\\ a.\sim\pi^*_{theorem1}(\cdot)\\ x.\sim P_{\mathcal{M}}(\cdot,\cdot)}}{\mathbb{E}} (r_0 + \gamma * r_1) \geq \underset{\substack{\mathcal{M}\sim\mathcal{D}\\ x_0\sim P_{x_0}\\ a.\sim\pi(\cdot)\\ x.\sim P_{\mathcal{M}}(\cdot,\cdot)}}{\mathbb{E}} (r_0 + \gamma * r_1)\ \forall \pi \in \Pi \quad . \tag{14}$$

Afterwards, we prove that the policy $\pi^*_{theorem1}$, generates for each time-step $t \geq 2$ a reward equal to R_{max} which is the maximum reward achievable, or written alternatively as:

$$\underset{\substack{\mathcal{M}\sim\mathcal{D}\\ x_0\sim P_{x_0}\\ a.\sim\pi^*_{bayes}(\cdot)\\ x.\sim P_{\mathcal{M}}(\cdot,\cdot)}}{\mathbb{E}} (\sum_{t=2}^{\infty} \gamma^t * r_t) = \sum_{t=2}^{\infty} \gamma^t * R_{max} \geq \underset{\substack{\mathcal{M}\sim\mathcal{D}\\ x_0\sim P_{x_0}\\ a.\sim\pi(\cdot)\\ x.\sim P_{\mathcal{M}}(\cdot,\cdot)}}{\mathbb{E}} (\sum_{t=2}^{\infty} \gamma^t * r_t)\ \forall \pi \in \Pi \quad . \tag{15}$$

By merging (14) and (15), we have that

$$\underset{\substack{\mathcal{M}\sim\mathcal{D}\\ x_0\sim P_{x_0}(\cdot)\\ a.\sim\pi_{theorem1}(\cdot)\\ x.\sim P_{\mathcal{M}}(\cdot,\cdot)}}{\mathbb{E}} (\sum_{t=0}^{\infty} \gamma^t * r_t) \geq \underset{\substack{\mathcal{M}\sim\mathcal{D}\\ x_0\sim P_{x_0}(\cdot)\\ a.\sim\pi(\cdot)\\ x.\sim P_{\mathcal{M}}(\cdot,\cdot)}}{\mathbb{E}} (\sum_{t=0}^{\infty} \gamma^t * r_t)\ \forall \pi \in \Pi$$

which proves the theorem.

$\triangleright$ *Part 1.* Let us now prove inequality (14). The first thing to notice is that for a policy to maximise expression (13), it only needs to satisfy two conditions for all x_0. The first one: to select an action a_1, which knowing the value of (x_0, a_0, r_0, x_1), maximises the expected value of r_1. We denote by $V_1(x_0, a_0, r_0, x_1)$ the maximum expected value of r_1 that can be obtained knowing the value of (x_0, a_0, r_0, x_1). The second one: to select an action a_0 knowing the value of x_0 that maximises the expected value of the sum $r_0 + \gamma V_1(x_0, a_0, r_0, x_1)$. We now show that the policy $\pi_{theorem1}$ satisfies these two conditions.

Let us start with the first condition that we check by analysing four cases, which correspond to the four cases a), b), c), d) of policy $\pi_{theorem1}$ for time step $t = 1$.

a) If $r_0 = 10$, the maximum reward that can be obtained, we are in a context where a_0 belongs to the target interval. It is easy to see that, by playing $a_1 = x_1 + a_0 - x_0$, we will obtain r_1 equal to 10. This shows that in case a) for time step $t = 1$, $\pi_{theorem1}$ maximises this expected value of r_1.

b) If $|r_0| > \alpha_{max} - (a_0 - x_0) \quad \wedge \quad a_0 - x_0 > 0$ and $r_0 \neq 10$ it is easy to see that the value of α to which the MDP corresponds can be inferred from (x_0, a_0, r_0) and that the action $a_1 = a_0 + r_0$ will fall in the middle of the target interval, leading to a reward of 10. Hence, in this case also, the policy $\pi_{theorem1}$ maximises the expected value of r_1.

c) If $|r_0| > \alpha_{max} - (x_0 - a_0) \quad \wedge \quad a_0 - x_0 < 0$ and $r_0 \neq 10$, we are also in a context where the value of α can be inferred directly from (x_0, a_0, r_0) and the action $a_1 = a_0 - r_0$ targets the centre of the target interval, leading to a reward of 10. Here again, $\pi_{theorem1}$ maximises the expected value of r_1.

d) When none of the three previous conditions is satisfied, a is not satisfied and so $x_1 = x_0$, we need to consider two cases: $(a_0 - x_0) \geq 0$ and $(a_0 - x_0) < 0$. Let us first start with $(a_0 - x_0) \geq 0$. In such a context, $\alpha \in \{a_0 - x_0 + r_0; a_0 - x_0 - r_0\} = \{a_0 - x_0 - |a_0 - x_0 - \alpha|, a_0 - x_0 + |a_0 - x_0 - \alpha|\}$ and where:

1) $P(\alpha = a_0 - x_0 - |a_0 - x_0 - \alpha||x_0, a_0, r_0, x_1) = 0.5$
2) $P(\alpha = a_0 - x_0 + |a_0 - x_0 - \alpha||x_0, a_0, r_0, x_1) = 0.5 \quad .$

Let us now determine the action a_1 that maximises $\hat{r}_1$, the expected value of r_1 according to $P(\alpha|x_0, a_0, r_0, x_1)$. Five cases, represented on Figure 1, have to be considered:

1) $a_1 < a_0 - |a_0 - x_0 - \alpha| - 1$. Here $\hat{r}_1 = a_1 - a_0$ and the maximum of $\hat{r}_1$ is equal to $-|a_0 - x_0 - \alpha| - 1$.
2) $a_1 \in [a_0 - |a_0 - x_0 - \alpha| - 1, a_0 - |a_0 - x_0 - \alpha| + 1]$. Here we have $\hat{r}_1 = \frac{1}{2}(10 + a_0 - |a_0 - x_0 - \alpha| - a_1)$ whose maximum over the interval is $5.5 - |a_0 - x_0 - \alpha|$ which is reached for $a_1 = a_0 + |a_0 - x_0 - \alpha| - 1$.
3) $a_1 \in [a_0 - |a_0 - x_0 - \alpha| + 1, a_0 + |a_0 - x_0 - \alpha| - 1]$. In this case $\hat{r}_1 = -|a_0 - x_0 - \alpha|$ and is independent from a_1.
4) $a_1 \in [a_0 + |a_0 - x_0 - \alpha| - 1, a_0 + |a_0 - x_0 - \alpha| + 1]$. The expected reward is $\hat{r}_1 = \frac{1}{2}(10 + a_0 - |a_0 - x_0 - \alpha| - a_1)$ whose maximum over the interval is $5.5 - |a_0 - x_0 - \alpha|$ which is reached for $a_1 = a_0 + |a_0 - x_0 - \alpha| + 1$.
5) $a_1 > a_0 + |a_0 - x_0 - \alpha| + 1$. In this case the expected reward is $\hat{r}_1 = a_0 - a_1$ and the maximum of $\hat{r}_1$ is equal to $-|a_0 - x_0 - \alpha| - 1$.

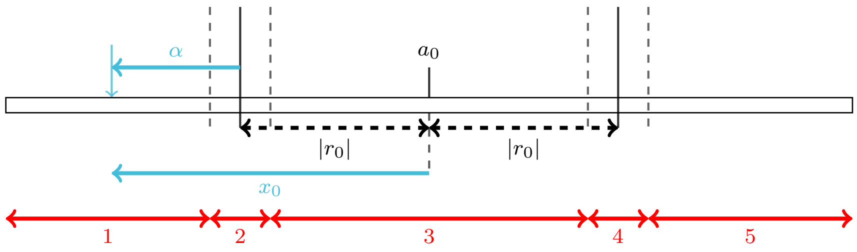

Fig 1. Graphical representation of the 5 different cases when playing a_1.

From 1), 2), 3), 4) and 5) one can see that, given the conditions considered here, an optimal policy can either play $a_1 = a_0 + |a_0 - x_0 - \alpha| - 1$ or $a_1 = a_0 - |a_0 - x_0 - \alpha| + 1$. In the following we will fix a_1 to $a_0 + |a_0 - x_0 - \alpha| + 1$ when $a_0 - x_0 \geq 0$. Let us also observe that the expected value of r_1 is equal to $5.5 - |a_0 - x_0 - \alpha|$. Up to

now in this item d), we have only considered the case where $(a_0 - x_0) > 0$. When $(a_0 - x_0) \leq 0$, using the same reasoning we reach the exact same expression for the optimal action to be played and for the maximum expected return of r_1. This is due to the symmetry that exists between both cases. Since $\pi_{theorem1}$ plays the action $a_1 = a_0 + r_0 + 1 = a_0 - |a_0 - x_0 - \alpha| + 1$ in the case d) at time step 1, it is straightforward to conclude that, in this case, it also plays an action that maximises the expected value of r_1.

Now that the first condition for $\pi_{theorem1}$ to maximise expression (13) has been proved, let us turn our attention to the second one. To this end, we will compute for each $x_0 \in \mathcal{X}$, the action $a_0 \in \mathcal{A}$ that maximises:

$$\mathop{\mathbb{E}}_{\substack{\alpha \sim \mathbb{U}_\alpha \\ x_1 \sim P_{\mathcal{M}(\alpha)}(x_0, a_0)}} (r_0 + \gamma * V_1(x_0, a_0, r_0, x_1)) \tag{16}$$

and show that this action coincide with the action taken by $\pi_{theorem1}$ for time step $t = 0$. First let us observe that for this optimisation problem, one can reduce the search space $\mathcal{A}$ to $[x_0 - \alpha_{max} + 1, x_0 + \alpha_{max} - 1] \subset \mathcal{A}$. Indeed, an action a_0 that does not belong to this latter interval would not give more information about α than playing $a_0 = x_0 - \alpha_{max} + 1$ or $x_0 + \alpha_{max} - 1$ and lead to a worse expected r_0. This reduction of the search space will be exploited in the developments that follow.

However, we should first remember that $\mathbb{U}_\alpha = \mathbb{U}[-\alpha_{max}, \alpha_{max}]$ and that the function $V_1(x_0, a_0, r_0, x_1)$ can be written as follows:

1. if $r_0 = 10$, V_1 is equal to $R_{max} = 10$
2. else if $|r_0| > \alpha_{max} - (a_0 - x_0) \quad \wedge \quad a_0 - x_0 > 0$ and $r_0 \neq 10$, then V_1 is equal to $R_{max} = 10$
3. else if $|r_0| > \alpha_{max} - (x_0 - a_0) \quad \wedge \quad a_0 - x_0 < 0$ and $r_0 \neq 10$, then V_1 is equal to $R_{max} = 10$
4. and otherwise V_1 is equal to $5.5 - |a_0 - x_0 - \alpha|$.

We note that the value of $V_1(x_0, a_0, r_0, x_1)$ does not depend on the state x_1, which allows us to rewrite expression (16) as follows:

$$\mathop{\mathbb{E}}_{\alpha \sim \mathbb{U}_\alpha} (r_0 + \gamma * V_1(x_0, a_0, r_0, x_1)) \tag{17}$$

and since the expectation is a linear operator:

$$(17) = \mathop{\mathbb{E}}_{\alpha \sim \mathbb{U}_\alpha} (r_0) + \gamma * \mathop{\mathbb{E}}_{\alpha \sim \mathbb{U}_\alpha} (V_1(x_0, a_0, r_0, x_1)) \quad . \tag{18}$$

Let us now focus on the second term of this sum:

$$\mathop{\mathbb{E}}_{\alpha \sim \mathbb{U}_\alpha} (V_1(x_0, a_0, r_0, x_1)) \quad . \tag{19}$$

We note that when $a_0 - x_0 \geq 0$ the function V_1 can be rewritten under the following form:

1. if $\alpha \in [-\alpha_{max}, 2*(a_0 - x_0) - \alpha_{max}[$, V_1 is equal to 10
2. else if $\alpha \in [2*(a_0 - x_0) - \alpha_{max}, a_0 - x_0 - 1]$, v_1 is equal to $5.5 + \alpha - (a_0 - x_0)$
3. else if $\alpha \in [a_0 - x_0 - 1, a_0 - x_0 + 1]$, V_1 is equal to 10
4. else if $\alpha \in]a_0 - x_0 + 1, \alpha_{max}]$, V_1 is equal to $5.5 - \alpha + (a_0 - x_0)$.

From here, we can compute the value of expression (19) when $a_0 - x_0 \geq 0$. We note that due to the symmetry that exists between the case $a_0 - x_0 \geq 0$ and $a_0 - x_0 \leq 0$, expression (19) will have the same value for both cases. Since we have:

$$(19) = \int_{-\infty}^{\infty} V_1 * p_\alpha * d\alpha$$

where p_α is the probability density function of α, we can write:

$$\begin{aligned}(19) &= \int_{-\alpha_{max}}^{\alpha_{max}} V_1 * \frac{1}{2*\alpha_{max}} d\alpha \\ &= \int_{-\alpha_{max}}^{2*(a_0-x_0)-\alpha_{max}} \frac{10}{2*\alpha_{max}} d\alpha + \int_{2*(a_0-x_0)-\alpha_{max}}^{a_0-x_0-1} \frac{5.5+\alpha-(a_0-x_0)}{2*\alpha_{max}} d\alpha \\ &+ \int_{a_0-x_0-1}^{a_0-x_0+1} \frac{10}{2*\alpha_{max}} d\alpha + \int_{a_0-x_0+1}^{\alpha_{max}} \frac{5.5-\alpha+(a_0-x_0)}{2*\alpha_{max}} d\alpha \quad .\end{aligned}$$

And thus, by computing the integrals, we have:

$$\begin{aligned}\mathop{\mathbb{E}}_{\alpha \sim \mathbb{U}_\alpha}(V_1) = -\frac{1}{2*\alpha_{max}}(a_0 - x_0)^2 + \frac{1}{\alpha_{max}}(\alpha_{max} + 4.5)*(a_0 - x_0) \\ + \frac{1}{\alpha_{max}}(5 + 5.5*\alpha_{max} - \frac{\alpha_{max}^2}{2}) \quad .\end{aligned}$$

Let us now analyse the first term of the sum in equation (18), namely $\mathop{\mathbb{E}}_{\alpha \sim \mathbb{U}_\alpha}(r_0)$.

We have that:

$$\mathop{\mathbb{E}}_{\alpha \sim \mathbb{U}_\alpha}(r_0) = \int_{-\infty}^{\infty} (r_0|x_0, a_0, \alpha) * p_\alpha * d\alpha$$

which can be rewritten as:

$$\mathop{\mathbb{E}}_{\alpha \sim \mathbb{U}_\alpha}(r_0) = \int_{-\alpha_{max}}^{\alpha_{max}} (r_0|x_0, a_0, \alpha) * \frac{1}{2*\alpha_{max}} d\alpha \quad .$$

Due to the reduction of the search space, we can assume that a_0 belongs to $[x_0 - \alpha_{max} + 1, x_0 + \alpha_{max} - 1]$, we can write:

$$\begin{aligned}\int_{-\alpha_{max}}^{\alpha_{max}} (r_0|x_0, a_0, \alpha) * \frac{1}{2*\alpha_{max}} d\alpha = \int_{-\alpha_{max}}^{a_0-x_0-1} \frac{\alpha-(a_0-x_0)}{2*\alpha_{max}} d\alpha \\ + \int_{a_0-x_0-1}^{a_0-x_0+1} \frac{10}{2*\alpha_{max}} d\alpha + \int_{a_0-x_0+1}^{\alpha_{max}} \frac{(a_0-x_0)-\alpha}{2*\alpha_{max}} d\alpha \quad .\end{aligned}$$

Given that $R_{max} = 10$, we have:

$$\mathop{\mathbb{E}}_{\alpha \sim \mathbb{U}_\alpha}(r_0) = \frac{-(a_0 - x_0)^2 + 21 - \alpha_{max}^2}{2 * \alpha_{max}}$$

and therefore:

$$\begin{aligned}(18) = &-\frac{1+\gamma}{2 * \alpha_{max}} * (a_0 - x_0)^2 + \frac{\gamma}{\alpha_{max}}(\alpha_{max} + 4.5) * (a_0 - x_0) \\ &+ \frac{1}{2 * \alpha_{max}}(21 - \alpha_{max}^2 + \gamma * (10 + 11 * \alpha_{max} - \alpha_{max}^2)) \quad .\end{aligned}$$

To find the action a_0 that maximises (16), one can differentiate (18) with respect to a_0:

$$\frac{d(18)}{d(a_0)} = -\frac{1}{\alpha_{max}} * (1+\gamma)(a_0 - x_0) + \frac{\gamma}{\alpha_{max}}(\alpha_{max} + 4.5) \quad .$$

This derivative has a single zero value equal to:

$$a_0 = \frac{\gamma * (\alpha_{max} + 4.5)}{1 + \gamma} + x_0 \quad .$$

It can be easily checked that it corresponds to a maximum of expression (16) and since it also belongs to the reduced search space $[x_0 - \alpha_{max} + 1, x_0 + \alpha_{max} - 1]$, it is indeed the solution to our optimisation problem. Since $\pi_{theorem1}$ plays this action at time $t = 0$, *Part 1* of this proof is now fully completed.

▷ *Part 2.* Let us now prove that the policy $\pi^*_{theorem1}$ generates for every $t \geq 2$ rewards equal to $R_{max} = 10$. We will analyse three different cases, corresponding to the three cases a), b) and c) of policy $\pi_{theorem1}$ for time step $t \geq 2$.

a) If $r_0 = 10$, we are in a context where a_0 belong to the target interval. It is straightforward to see that, by playing $a_t = x_t + a_0 - x_0$, the action played by $\pi_{theorem1}$ in this case, we will get a reward r_t equal to 10.

b) If $r_1 = 10$ and $r_0 \neq 10$, one can easily see that playing action $a_t = x_t + a_1 - x_1$, the action played by $\pi_{theorem1}$, will always generate rewards equal to 10.

c) If $r_0 \neq 10$ and $r_1 \neq 10$, it is possible to deduce from the first action a_0 that the MDP played corresponds necessarily to one of these two values for α: $\{a_0 - x_0 + r_0; a_0 - x_0 - r_0\}$. Similarly, from the second action played, one knows that α must also stand in $\{a_1 - x_1 + r_1; a_1 - x_1 - r_1\}$. It can be proved that because $a_0 \neq a_1$ (a property of our policy $\pi_{theorem1}$), the two sets have only one element in common. Indeed if these two sets had all their elements in common, either this pair of equalities would be valid:

$$\begin{aligned} a_0 - x_0 + r_0 &= a_1 - x_1 + r_1 \\ a_0 - x_0 - r_0 &= a_1 - x_1 - r_1 \end{aligned}$$

or this pair of equalities would be valid:

$$a_0 - x_0 + r_0 = a_1 - x_1 - r_1$$
$$a_0 - x_0 - r_0 = a_1 - x_1 + r_1 \quad .$$

By summing member by member the two equations of the first pair, we have:

$$a_0 - x_0 = a_1 - x_1 \quad .$$

Taking into account that $x_0 = x_1$ because none of the two actions yielded a positive reward, it implies that $a_0 = a_1$, which results in a contradiction. It can be shown in a similar way that another contradiction appears with the second pair. As a result the intersection of these two sets is unique and equal to α. From here, it is straightforward to see that in this case c), the policy $\pi_{theorem1}$ will always generate rewards equal to R_{max}.

■

From Theorem 1, one can easily prove the following theorem.

Theorem 2 *The value of expected return of a Bayes optimal policy for benchmark 1 is equal to* $\frac{3*\gamma^2*(\alpha_{max}+4.5)^2}{2*\alpha_{max}*(1+\gamma)} + \frac{21+\alpha_{max}^2+\gamma*(10+11*\alpha_{max}-\alpha_{max}^2)}{2*\alpha_{max}} + \frac{\gamma^2}{1-\gamma} * 10$.

Proof The expected return of a Bayes optimal policy can be written as follows:

$$\mathop{\mathbb{E}}_{\substack{\mathcal{M}\sim\mathcal{D}\\ x_0\sim P_{x_0}\\ a_\cdot\sim\pi^*_{bayes}(\cdot)\\ x_\cdot\sim P_{\mathcal{M}}(\cdot,\cdot)}} \sum_{t=0}^{1} \gamma^t * r_t + \mathop{\mathbb{E}}_{\substack{\mathcal{M}\sim\mathcal{D}\\ x_0\sim P_{x_0}\\ a_\cdot\sim\pi^*_{bayes}(\cdot)\\ x_\cdot\sim P_{\mathcal{M}}(\cdot,\cdot)}} \sum_{t=2}^{\infty} \gamma^t * r_t \quad .$$

From the proof of Theorem 1, it is easy to see that:

1. $\mathop{\mathbb{E}}_{\substack{\mathcal{M}\sim\mathcal{D}\\ x_0\sim P_{x_0}\\ a_\cdot\sim\pi^*_{bayes}(\cdot)\\ x_\cdot\sim P_{\mathcal{M}}(\cdot,\cdot)}} \sum_{t=0}^{1} \gamma^t * r_t = \frac{3*\gamma^2*(\alpha_{max}+4.5)^2}{2*\alpha_{max}*(1+\gamma)} + \frac{21+\alpha_{max}^2+\gamma*(10+11*\alpha_{max}-\alpha_{max}^2)}{2*\alpha_{max}}$
2. $\mathop{\mathbb{E}}_{\substack{\mathcal{M}\sim\mathcal{D}\\ x_0\sim P_{x_0}\\ a_\cdot\sim\pi^*_{bayes}(\cdot)\\ x_\cdot\sim P_{\mathcal{M}}(\cdot,\cdot)}} \sum_{t=2}^{\infty} \gamma^t * r_t = \frac{\gamma^2}{1-\gamma} 10$

which proves Theorem 2. ■

References

1. Williams RJ. Simple statistical gradient-following algorithms for connectionist reinforcement learning. Machine learning. 1992;8(3-4):229–256.

2. Schulman J, et al. High-Dimensional Continuous Control Using Generalized Advantage Estimation. CoRR. 2015;abs/1506.02438.

3. Schulman J, et al. Trust region policy optimization. In: International Conference on Machine Learning; 2015. p. 1889–1897.

4. Schulman J, et al. Proximal Policy Optimization Algorithms. CoRR. 2017;abs/1707.06347.

5. Coady P. AI Gym workout;. `https://learningai.io/projects/2017/07/28/ai-gym-workout.html`.

6. Kingma DP, et al. ADAM: A Method for Stochastic Optimization. CoRR. 2014;abs/1412.6980.

7. Werbos PJ, et al. Backpropagation through time: what it does and how to do it. Proceedings of the IEEE. 1990;78(10):1550–1560.
